# Dengue Virus Impairs Mitochondrial Fusion by Cleaving Mitofusins

**DOI:** 10.1371/journal.ppat.1005350

**Published:** 2015-12-30

**Authors:** Chia-Yi Yu, Jian-Jong Liang, Jin-Kun Li, Yi-Ling Lee, Bi-Lan Chang, Chan-I Su, Wei-Jheng Huang, Michael M. C. Lai, Yi-Ling Lin

**Affiliations:** 1 Department of Medical Laboratory Science and Biotechnology, National Cheng Kung University, Tainan, Taiwan; 2 Center of Infectious Disease and Signaling Research, National Cheng Kung University, Tainan, Taiwan; 3 Institute of Biomedical Sciences, Academia Sinica, Taipei, Taiwan; 4 Department of Microbiology and Immunology, National Cheng Kung University, Tainan, Taiwan; 5 Institute of Molecular Biology, Academia Sinica, Taipei, Taiwan; 6 China Medical University, Taichung, Taiwan; 7 Genomics Research Center, Academia Sinica, Taipei, Taiwan; Icahn School of Medicine at Mount Sinai, UNITED STATES

## Abstract

Mitochondria are highly dynamic subcellular organelles participating in many signaling pathways such as antiviral innate immunity and cell death cascades. Here we found that mitochondrial fusion was impaired in dengue virus (DENV) infected cells. Two mitofusins (MFN1 and MFN2), which mediate mitochondrial fusion and participate in the proper function of mitochondria, were cleaved by DENV protease NS2B3. By knockdown and overexpression approaches, these two MFNs showed diverse functions in DENV infection. MFN1 was required for efficient antiviral retinoic acid-inducible gene I–like receptor signaling to suppress DENV replication, while MFN2 participated in maintaining mitochondrial membrane potential (MMP) to attenuate DENV-induced cell death. Cleaving MFN1 and MFN2 by DENV protease suppressed mitochondrial fusion and deteriorated DENV-induced cytopathic effects through subverting interferon production and facilitating MMP disruption. Thus, MFNs participate in host defense against DENV infection by promoting the antiviral response and cell survival, and DENV regulates mitochondrial morphology by cleaving MFNs to manipulate the outcome of infection.

## Author Summary

Dengue virus (DENV) threatens billions of people worldwide but no licensed vaccine or therapeutics is currently available. Knowing more details of DENV pathogenesis, such as antagonism of host immunity and cell death induction, may provide important clues to fight against this thorny disease. Incoming studies showed that mitochondria are not only energy providers but also regulators of antiviral signaling pathways including interferon innate immunity and cell death induction. Furthermore, the normal functions of mitochondrion can be regulated by its dynamics through constant fusion and fission. In this study, we found that DENV infection caused an impairment of mitochondrial fusion and the two key players, mitofusin-1 and -2, mediating the fusion processes in mitochondrial dynamics, were cleaved by DENV protease. Cleaving mitofusins altered mitochondrial morphology, attenuated antiviral responses, and facilitated cell death upon DENV infection. Thus, DENV could manipulate mitochondrial functions by taking over mitochondrial dynamics to benefit viral replication, and the viral protease of DENV may serve as a virulence factor besides being an enzyme responsible for the processing of viral proteins.

## Introduction

Mitochondria, the powerhouse of cells, participate in various cellular events, such as ATP production, fatty acid synthesis, calcium homeostasis, and apoptosis induction [1,2]. Their roles in cell signaling are also emerging: mitochondria can sense perturbations of intracellular homeostasis, then regulate and transduce signaling responses, especially during stressful conditions [3,4]. The identification of mitochondrial antiviral signaling (MAVS), a mitochondrial outer-membrane protein functioning as the adaptor of retinoic acid-inducible gene I (RIG-I)–like receptors (RLRs), revealed a link between mitochondria and antiviral innate immunity. Cytosolic viral RNA recognized by RLRs can activate MAVS and recruit various signaling molecules to transduce the downstream pathways, such as type I interferon (IFN) production [5–8] and cell death induction [9,10], two major cellular events controlling viral infection.

Mitochondria are highly dynamic double-membrane organelles, and their shapes change continually via the combined actions of fusion, fission and trafficking [11,12]. These dynamic events play critical roles in maintaining functional mitochondria because fusion promotes complementation of damaged mitochondria and fission creates new mitochondria [13,14]. Therefore, a balanced mitochondrial dynamics keeps mitochondria in good health and disturbing such physiological balance would contribute to diseases, such as abnormal brain development, autosomal dominant optic atrophy and Charcot-Marie-Tooth type 2A [15,16].

Two mitofusin proteins, MFN1 and MFN2, located on the mitochondrial outer membrane, mediate tethering and fusion of mitochondria [3,4,17]. Human MFN1 and MFN2 share 63% protein sequence identity and have the same relevant functional domains: a GTPase domain at the N-terminus, two coiled-coil domains (HR1 and HR2), and a transmembrane (TM) domain at the C-terminus [18]. MFN2 but not MFN1 contains a proline-rich region, and MFN2 is also present in the endoplasmic reticulum (ER) in addition to mitochondria [19]. Both MFNs mediate mitochondrial outer-membrane fusion by tethering of two adjacent mitochondria membranes with their HR2 domains, followed by GTP-required docking of both membranes before final fusion.

Accumulated findings demonstrated that antiviral RLR signaling can be regulated by the dynamics of mitochondria [4,20–22]. Fibroblasts deficient in both MFNs showed impaired induction of RLR-induced antiviral responses [23]. MFN2 has been shown to interact with MAVS and suppress MAVS activating the IFNβ promoter [24]. Other reports also showed that MFN1 interacts with MAVS and mitochondrial fusion is required for efficient RLR signaling [25,26]. Thus, MFNs interact with MAVS and modulate RLR signaling, but the detailed involvement of these two MFNs in viral infection is largely unclear.

Dengue virus (DENV) is an enveloped flavivirus with a positive-sense RNA genome encoding a polyprotein. DENV infection in humans can cause diseases ranging from mild self-limited dengue fever to life-threatening dengue hemorrhagic and dengue shock syndrome [27]. The virus is transmitted by mosquitos, with possibly 390 million DENV infections every year [28]. So far, we lack an approved DENV vaccine or anti-DENV drug. Cells mainly sense DENV infection by two cytosolic RLRs, RIG-I and MDA5, then induce IFN production in a MAVS-dependent pathway [29,30]. In cells or animals with deleted MAVS, DENV-triggered IFN induction is significantly reduced, while DENV-induced mitochondrial membrane potential (MMP) disruption [10], cytopathic effects [10] and murine mortality [31] are attenuated. Thus, MAVS is involved in two cellular antiviral events, IFN induction and cell death, and contributes to the control and pathogenesis of DENV infection. To antagonize MAVS-mediated antiviral signaling, MAVS protein is cleaved by virus proteases such as that of hepatitis C virus (HCV) [6,32] and picornaviruses [33,34], and by cellular caspase in the cases of DENV infection [10], while MAVS-associated cofactor MITA/STING is cleaved by the DENV protease NS2B3 [35,36]. Thus, MAVS plays important roles in triggering the host defense against DENV infection, and DENV has evolved ways to counteract the MAVS-mediated signaling pathway.

In this study, we explored the relationship between DENV and mitochondrial dynamics and found that DENV infection can disrupt mitochondrial fusion by cleaving MFNs. Using cells with inducible expression of MFN1 or MFN2 and cells with MFN1 or MFN2 knockdown, we found that MFN1 was required for efficient RLR signaling, whereas MFN2 reduced the cell death triggered by DENV infection. Thus, we provide the first example of viral modulating of mitochondria dynamics by cleaving MFNs and identified two new cellular substrates of DENV protease. Our study shows that manipulating mitochondrial dynamics by viral protease could be one of the mechanisms contributing to DENV pathogenesis.

## Results

### DENV infection attenuates mitochondrial dynamics

**Fig 1:**
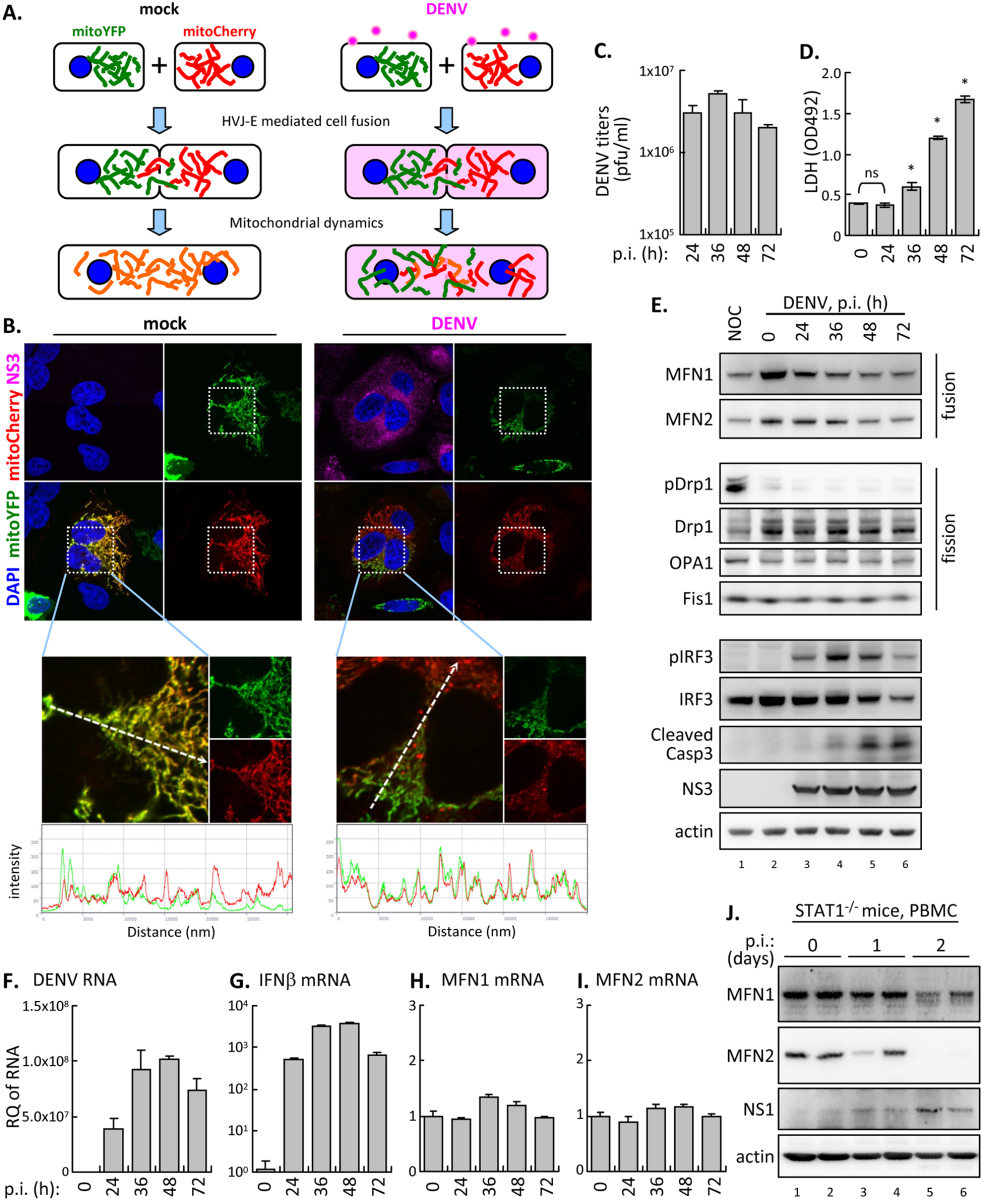
DENV infection impairs mitochondrial dynamics. (A and B) Diagram (A) and the results (B) of mitochondria intermixing assay. Mitochondria of A549 cells were labeled by transfection with mitoYFP or mitoCherry to distinguish the origin of each mitochondrion. Cells were mock or infected with DENV serotype 2 (multiplicity of infection [moi] 10) for 24 h before HVJ-E-mediated cell fusion. The fused cell hybrids with or without DENV infection were magnified and analyzed for green and red fluorescence intensity by use of ZEN lite 2011 (Carl Zeiss MicroImaging GmbH). Green: mitoYFP; red: mitoCherry; magenta: DENV NS3. (C to I) Time course study of DENV infection in human A549 cells with moi 10 for the indicated hours. Culture supernatants were harvested for virus titration (C) and release of lactic dehydrogenase (LDH) (D). Cell lysates were harvested for western blot analysis with the indicated antibodies (E) and RT-qPCR (F to I) for the indicated genes. Data are mean ± SD (*n = 3* per group). The data for LDH release was compared by one way ANOVA and Bonferroni multiple-comparison test with use of Prism 5 (GraphPad; La Jolla, CA, USA). ns, no significance; *, p<0.05. Nocodazole treatment (NOC; 100 ng/ml for 16 h) served as positive control to induce Drp1 phosphorylation at S616 residue. RQ, relative quantification. (J) STAT1^-/-^ mice were inoculated with DENV (serotype 2, strain NGC-N) by an intraperitoneal plus intracerebral route. The peripheral blood mononuclear cells (PBMC) were isolated on day 0, 1, and 2 as indicated (*n* = 2 for each time point) and then sampled for western blot analysis with the indicated antibodies.

To explore whether DENV can affect mitochondrial dynamics, we performed a mitochondrial intermixing experiment, which has been used to investigate mitochondrial fusion/fission events by fusing two individual cells [37,38]. We established two stable A549 cell lines with either mitochondria-targeted YFP (mitoYFP) or RFP (mitoCherry) and fused them by using an HVJ Envelope Cell fusion kit (Fig 1A). In mock-infected cells, continuous mitochondrial fusion/fission led to an even redistribution and colocalization of mitoYFP and mitoCherry in the fused cells, whereas much less colocalization of mitoYFP and mitoCherry was noted in the fused DENV-infected cells (Fig 1B) at 24 h post infection (p.i.) when DENV replicated to high titer (Fig 1C) but without significant cytotoxicity (Fig 1D). The delayed redistribution of mitochondria might be resulted from reduced mobility, enhanced fission or suppressed fusion. We found no obvious difference in the mitochondrial movement between mock- and DENV-infected cells by the live-confocal microscopy analysis (S1 Movie and S1 Fig). We also checked the phosphorylation level of the fission-related dynamin-related protein 1 (Drp1) at Ser616 (pDrp1), which was induced by hepatitis B virus (HBV) and HCV to promote mitochondrial fission [39,40], as well as the endogenous protein levels of several mitochondria fusion/fission regulators. Levels of fission-related proteins, Drp1, pDrp1, optic atrophy 1 (OPA1) and mitochondrial fission 1 (Fis1) were similar during the course of DENV infection (Fig 1E and S2 Fig), while reduced fusion-related MFN1 and MFN2 proteins were detected in DENV-infected cells (Fig 1E). The mRNA levels of MFN1 and MFN2 remained constant in DENV-infected cells when viral RNA and IFNβ were induced exponentially (Fig 1F–1I), so the downregulation of MFNs proteins likely occurred at post-transcriptional level. Moreover, MFN2 protein levels were reduced in the peripheral blood mononuclear cells (PBMC) isolated from STAT1-deficient mice during the course of DENV infection (Fig 1J). Overall, despite the role of mitochondrial fission-related molecules cannot be completely excluded, our data indicated that mitochondrial fusion might be affected in DENV-infected cells.

### DENV infection suppresses MFN1-mediated mitochondrial fusion

**Fig 2:**
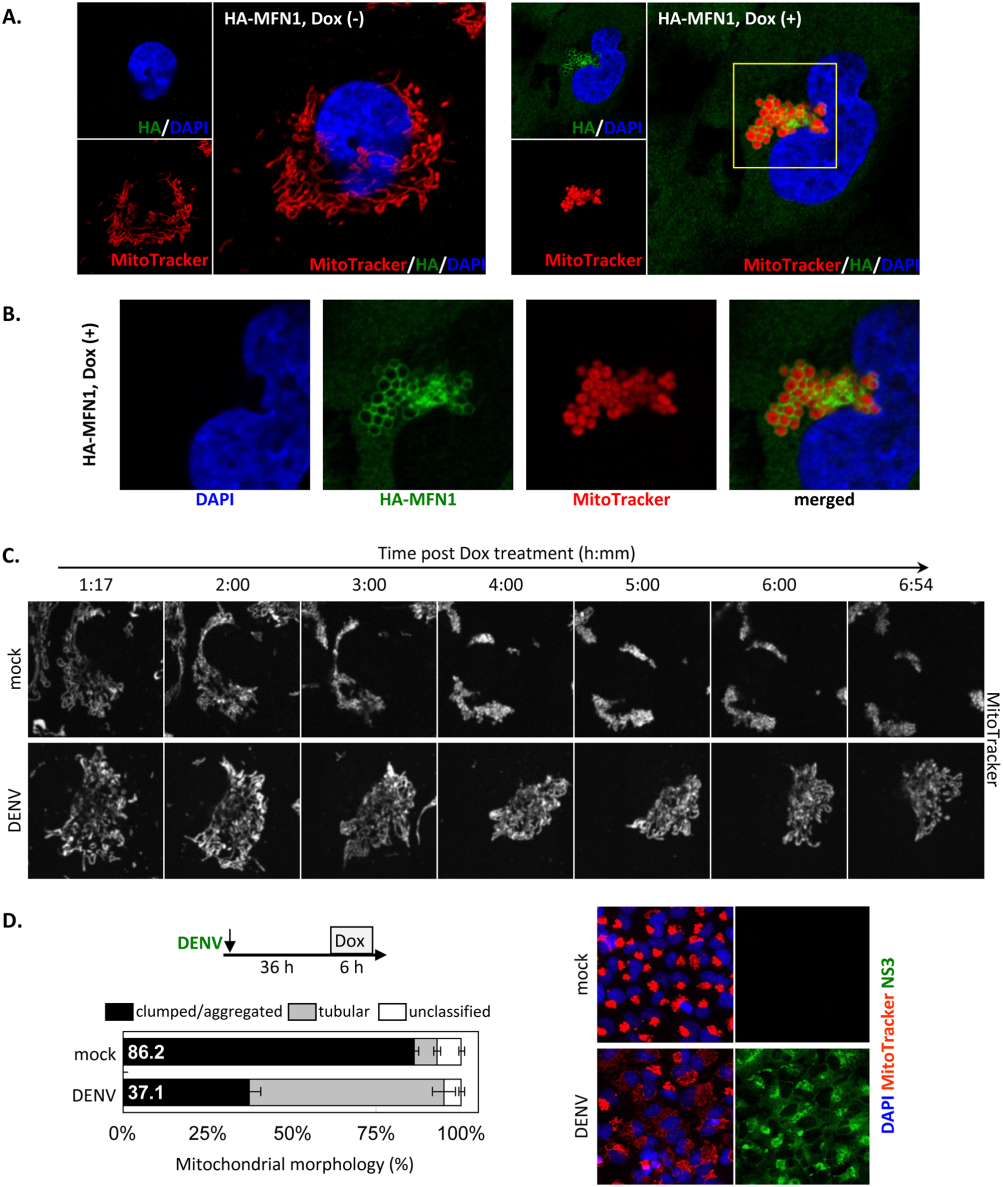
DENV infection suppresses MFN1-mediated mitochondrial dynamics. (A) Anti-HA and MitoTracker staining of intracellular localization of ectopic-expressed MFN1 and mitochondrial morphology in A549^+on^/HA-MFN1 cells treated with or without Dox (1 μg/ml, 24 h). (B) High-power analysis of MFN1-overexpressing A549 cells as described in A. The magnified area is marked by square. (C) A549^+on^/HA-MFN1 cells were infected with DENV serotype 2 (moi 10) for 36 h and then stained with MitoTracker for 30 min before Dox induction. The mitochondrial dynamics of mock- and DENV-infected cells were photographed side by side every 30 sec continuously after Dox treatment. A representative cell for each condition is shown. (D) A549^+on^/HA-MFN1 cells were infected, stained, and treated with Dox as in panel C. Cells were fixed after 6 h of Dox treatment and then mitochondrial morphologic features were quantified by analyzing immunofluorescent images (one set of random selected fields in each group is shown at the right) from 49 individual fields by high-content analysis (ImageXpress Micro Imaging XL System, Molecular Devices). Data at left are mean ± SD from 49 individual fields of each sample.

To ask whether mitochondrial fusion is blocked by DENV infection, we overexpressed MFN1 to induce mitochondria hyperfusion and aggregated/grape-like cluster morphology [41,42]. To express MFN1 in a controlled manner, we established an A549^+on^ cell line that expressed an advanced Tet-On transactivator, which can turn on the Tet-responsive element (TRE)-tight promoter in the presence of doxycycline (Dox). We then transduced A549^+on^ cells with lentivirus expressing HA-tagged MFN1 under the control of TRE-tight promoter. With Dox treatment, the stable A549^+on^/HA-MFN1 cells expressed HA-MFN1 (Fig 2A) around mitochondria (Fig 2B) as previously reported [41,42]. Furthermore, distinct mitochondria morphologic features were seen in cells with or without MFN1 induction—clumped/aggregated versus filamentous/tubular mitochondria—as expected (Fig 2A).

We then addressed the potential influence of DENV infection on MFN1-triggered mitochondrial hyperfusion. A549^+on^/HA-MFN1 cells were mock-infected or infected with DENV for 36 h before Dox treatment. Clumped mitochondria aggregations were apparent within 6 h of Dox treatment in mock-infected MFN1-overexpressing cells, whereas mitochondria remained pleomorphic in DENV-infected cells during the same time frame (Fig 2C and S2 Movie). Quantification by high-content analysis confirmed the microscopy observations: the proportion of cells with clumped mitochondria decreased from 86.2% to 37.1% when compared the mock- to DENV-infected cells (Fig 2D). Thus, our data suggested that DENV can block MFN1-mediated mitochondria fusion.

### MFNs protect cells from DENV-induced cell death

**Fig 3:**
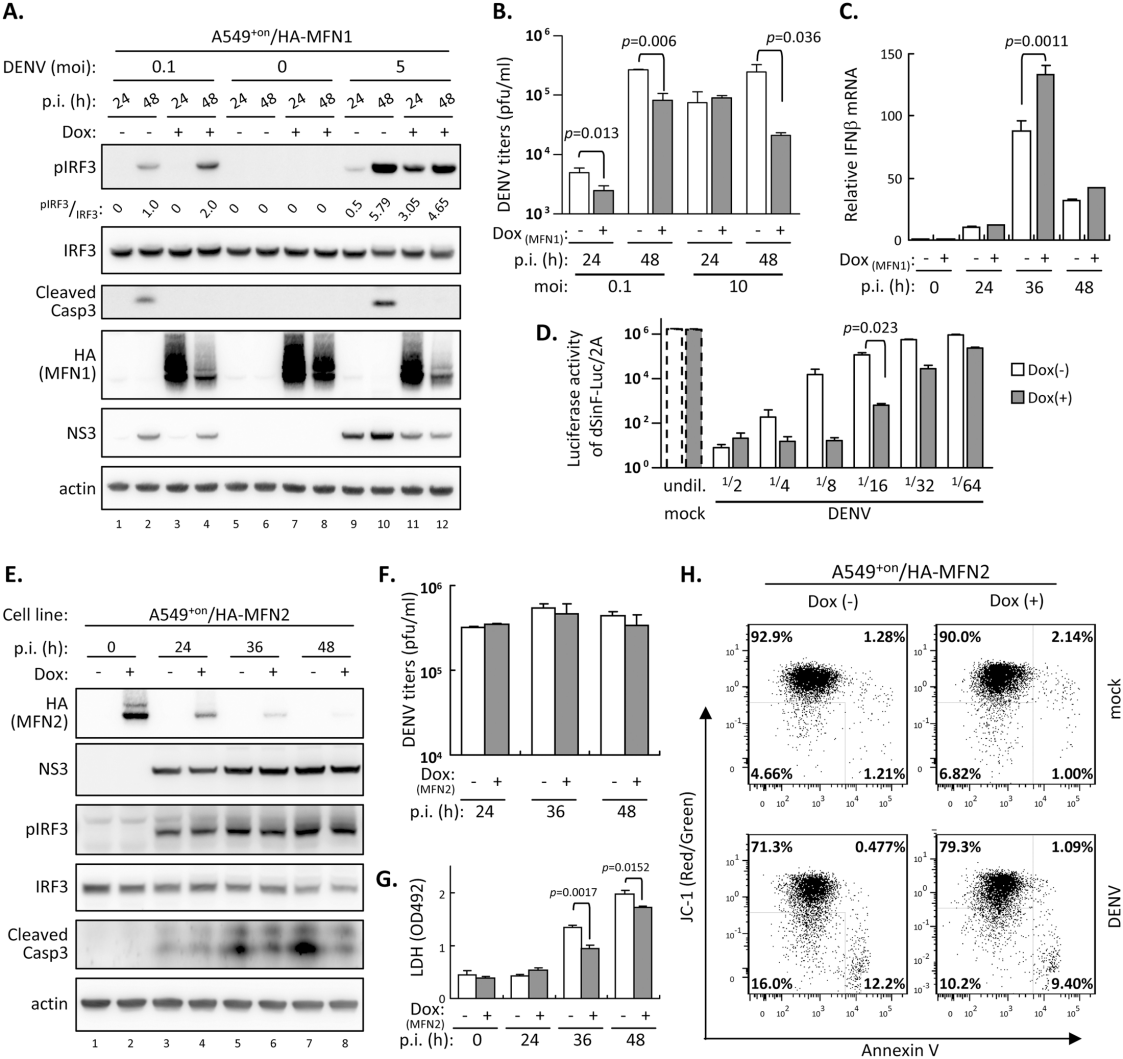
Effects of MFN1 or MFN2 overexpression on DENV infection. (A) Western blot analysis of A549^+on^/HA-MFN1 cells induced with Dox (1 μg/ml) or not for 18 h, then infected with DENV serotype 2 for 24 or 48 h by the indicated moi. The relative ratios of band intensity were quantified by ImageJ. p.i. (h): hours post infection. (B) DENV plaque-forming assay of culture supernatants from A549^+on^/HA-MFN1 cells cultured with or without Dox (1 μg/ml) for 18 h, then infected with DENV (moi 0.1 or 10) as indicated. Data in panels B-D and F-G are mean ± SD (*n = 3* per group) and were compared by two-tailed Student’s *t* test. (C) RT-qPCR analysis of IFNβ mRNA expression at indicated time point in DENV-infected (moi 10) A549^+on^/HA-MFN1 cells with or without an 18 h-Dox-pretreatment. (D) Analysis of antiviral activity against dSinF-Luc/2A virus in culture media from A549^+on^/HA-MFN1 cells with DENV infection (moi 5, 48 h) with or without 18-h Dox pretreatment. (E) Western blot analysis of A549^+on^/HA-MFN2 cells induced with Dox (1 μg/ml, 18 h) or not, then infected with DENV (moi 10) for the indicated time. (F and G) Analysis of the culture supernatants derived from panel E for DENV titer (F) and LDH release (G). (H) Flow cytometry of A549^+on^/HA-MFN2 cells double stained with JC-1 and annexin V. Cells were infected with DENV (moi 5) for 48 h with or without 18-h Dox pre-treatment. Decreased red/green fluorescence ratio of JC-1 represents disrupted MMP.

We next examined the effects of MFN1 overexpression on DENV infection. DENV-infected A549^+on^/HA-MFN1 cells with Dox pre-treatment showed higher IRF3 phosphorylation (pIRF3), an indicator of RLR signaling activation, and lower viral production when compared to cells without Dox treatment (Fig 3A and 3B). Higher IFNβ mRNA expression was noted in Dox-treated MFN1-overexpressing cells (Fig 3C). We further used an IFN-sensitive recombinant sindbis virus containing a *firefly* luciferase reporter gene (dSinF-Luc/2A) [35,43] to assess the antiviral activity. The culture supernatants from MFN1-overexpressing cells suppressed the replication of dSinF-Luc/2A to a greater extent than the Dox (-) control (Fig 3D), indicating higher antiviral activity in the MFN1-overexpressing cells. MFN1-overexpression also blocked the DENV-triggered activation of apoptotic marker caspase 3 (Fig 3A), probably because of reduced DENV replication. To ascertain whether MFN1 and MFN2 share the same features in DENV infection, A549^+on^/HA-MFN2 cells were established by lentivirus transduction as described for MFN1. Interestingly, MFN2 overexpression did not affect IRF3 phosphorylation, DENV viral protein expression (Fig 3E) or viral progeny production (Fig 3F), but DENV-induced cytopathic effects, measured by caspase 3 activation (Fig 3E), LDH release (Fig 3G) and MMP disruption, which initiates intrinsic route of apoptosis (Fig 3H; attenuated from 16 + 12.2 = 28.2% to 10.2 + 9.4 = 19.6%), were less evident in Dox-treated A549^+on^/HA-MFN2 cells. Therefore, MFN1 facilitated efficient RLR signaling and MFN2 alleviated MMP disruption during DENV infection. These data suggested that MFN1 and MFN2 play diverse roles in DENV infection despite that they share structural and functional similarity.

### DENV protease NS2B3 cleaves MFNs

**Fig 4:**
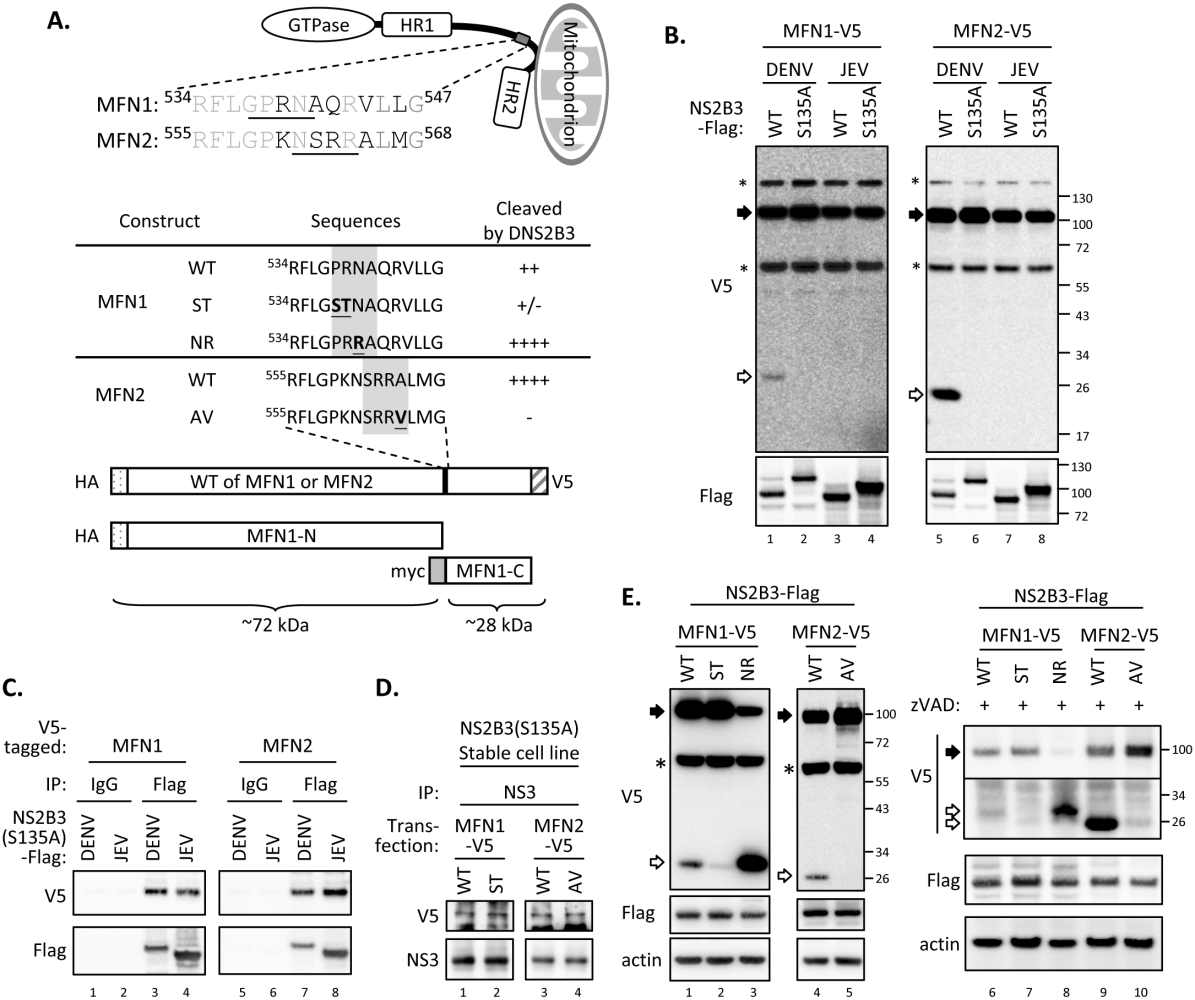
DENV cleaves human MFN1 and MFN2. (A) Alignment of amino acid sequences surrounding the MFN1 and MFN2 cleavage sites of DENV protease. The wild-type (WT) and mutated MFNs used in this study and the relative position of each domain are illustrated. (B) Western blot analysis of the cotransfection of C-terminal V5-tagged human MFN1 or MFN2 with Flag-tagged WT or mutated viral protease of DENV or JEV in A549 cells for 24 h. WT: wild-type; S135A: protease-dead mutant. (C) Coimmunoprecipitation analysis of A549 cells cotransfected with V5-tagged MFN1 or MFN2 with Flag-tagged NS2B3(S135A) of DENV or JEV for 24 h. (D) Coimmunoprecipitation analysis of A549 cells expressing S135A mutated DENV NS2B3 transfected with V5-tagged WT or mutated MFN1 or MFN2 for 24 h. (E) Western blot analysis of Flag-tagged DENV protease NS2B3 cotransfected with the indicated (WT or mutant) C-terminal V5-tagged MFNs constructs in A549 cells in the absence (lane 1–5) or presence (lane 6–10) of pan-caspase inhibitor (zVAD; 100 μM) for 24 h. Filled arrow: full-length; open arrow: cleaved product; star: non-specific band.

We noted protein reduction of endogenous and overexpressed MFNs with DENV infection (Figs 1E, 3A and 3E), so we explored whether MFN proteins were downregulated by DENV. We found several potential DENV protease-cleavage recognition sequences, two basic residues followed by a small amino acid [44–47], in human MFN1 and MFN2 proteins (Fig 4A). To test whether DENV protease can cleave MFNs, we cotransfected cells with Flag-tagged DENV NS2B3 plus C-terminal V5-tagged MFN1 or MFN2 for western blot analysis. Besides the full-length MFNs (~100 kDa), smaller protein bands of ~28 kDa were detected by anti-V5 antibody in cells with wild-type (WT) but not protease-dead (S135A) DENV NS2B3 (Fig 4B). The protease from another flavivirus, Japanese encephalitis virus (JEV), failed to cleave MFNs (Fig 4B, lane 3 and 7), even though they share the same cleavage recognition sequences [48] and both of them can physically interact with MFNs (Fig 4C), for unknown reasons.

**Fig 5:**
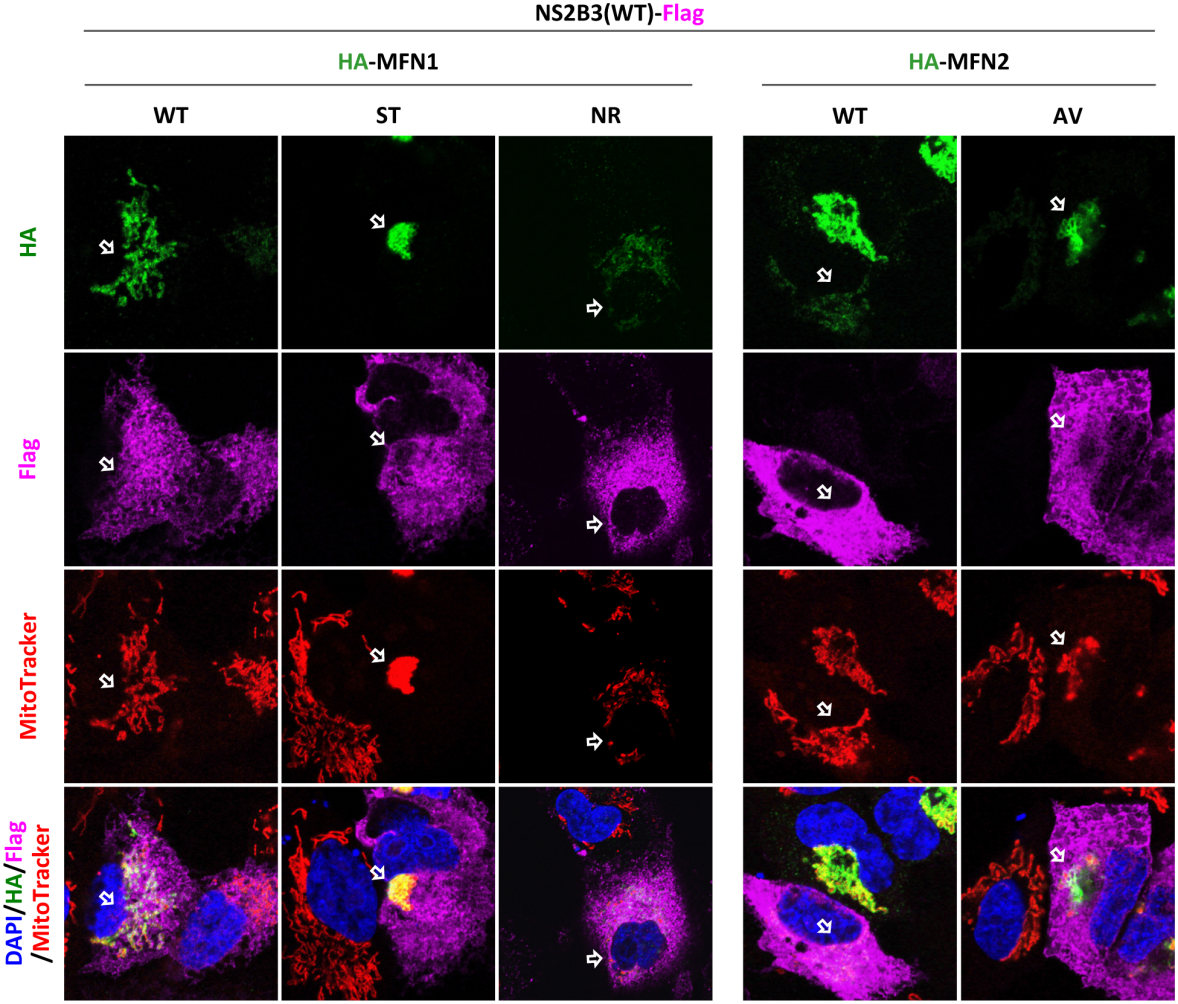
Suppression of MFN-triggered mitochondrial hyperfusion can be attributed to cleavage by DENV protease. Confocal microscopy of A549 cells cotransfected with Flag-tagged DENV protease and the indicated MFN constructs for 24 h. Arrows indicate the cells expressing both Flag-tagged DENV protease and HA-tagged MFN. Green: anti-HA; magenta: anti-Flag; red: MitoTracker; blue: DAPI.

Based on the sizes of the cleavage products (Fig 4B), we predicted the positions of ^538^PRN↓A^541^ in MFN1 and ^562^SRR↓A^565^ in MFN2 are the most likely cleaved sites by DENV protease NS2B3 (Fig 4A). To verify that the smaller protein bands are indeed the cleavage products at the predicted sites, we created the MFN1-ST mutant with potential cleavage sequences changed from ^538^ PRN↓A^541^ to ^538^ STN↓A^541^ and the MFN2-AV mutant with cleavage consensus sequences ^562^SRR↓A ^565^ changed to ^562^SRR↓V ^565^. Despite the mutant MFNs still bound with DENV protease (Fig 4D), much less cleavage products were seen in the MFN1-ST and MFN2-AV mutants (Fig 4E, lane 2 and 5). Because MFN1 ^538^PRN↓A^541^ is not a typical cleavage site for DENV protease, we created another mutant MFN1-NR, which contains a classical DENV protease cleavage site at ^538^PRR↓A^541^. As expected, MFN1-NR mutant was cleaved efficiently by DENV protease NS2B3 (Fig 4E, lane 3). These cleavage events were not affected by a pan-caspase inhibitor zVAD-fmk (Fig 4E, lane 6–10), indicating that caspases are not involved in the cleavage of MFNs by DENV protease. Furthermore, the mitochondrial hyperfusion triggered by dengue protease-sensitive WT MFNs and MFN1-NR mutant was attenuated in cells coexpressing DENV NS2B3(WT) (Fig 5) but not in cells with protease-dead NS2B3(S135A) (S3 Fig); whereas the hyperfusion triggered by the protease-resistant MFN1-ST or MFN2-AV was not affected by DENV NS2B3(WT) coexpression (Fig 5). Thus, the cleavage of MFNs by dengue protease hampered their function on mitochondrial fusion.

**Fig 6:**
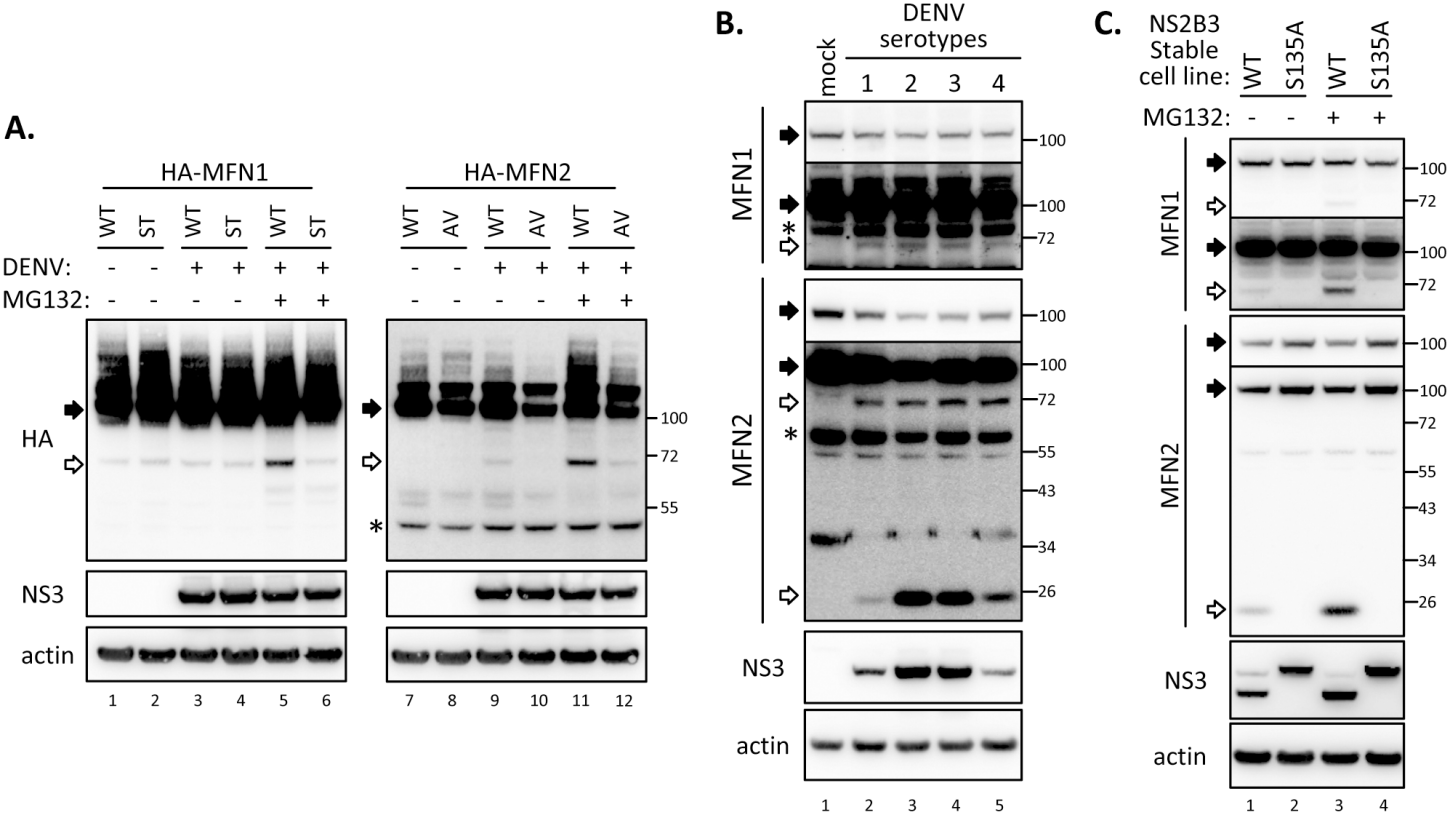
The cleaved MFN fragments were further degraded by host proteasome machinery. (A) Western blot analysis of A549^+on^/MFN1 (lane 1–6) and /MFN2 (lane 7–12) cells with Dox treatment (all lanes) and DENV infection (lane 3–6 and 9–12). The cells were infected with DENV serotype 2 (moi 5) for 24 h, then incubated in culture media containing Dox (1 μg/ml) with (lanes 5–6 and 11–12) or without MG132 (0.5 μM) for 16 h. (B) Western blot analysis of four different serotypes DENV-infected A549 cells incubated with MG132-containing medium. (C) Western blot analysis of endogenous MFN1 and 2 cleavage in A549 stable cells overexpressing WT or protease-dead mutant (S135A) of DENV NS2B3 with or without 18-h treatment with MG132 (0.5 μM). Filled arrow: full-length; open arrow: cleaved product; star: non-specific band. The longer exposure images for endogenous MFN1 and 2 signals are also shown in panels B and C.

We next addressed whether MFNs can be cleaved by DENV infection. In cells expressing N-terminal HA-tagged MFN1-WT but not the -ST mutant, anti-HA antibody revealed a cleavage product (~72 kDa) when MG132 was added to block proteasome-mediated protein degradation (Fig 6A, lanes 5–6). Similarly, the ~72 kDa cleaved fragment of MFN2 was evident in cells with MFN2-WT but not -AV mutant upon DENV infection with MG132 treatment (Fig 6A, lanes 11–12). Thus, our results suggested that DENV targets both MFNs by its viral protease and the cleaved MFN fragments were further degraded by host proteasome machinery. Moreover, endogenous MFN1 and 2 can be cleaved by four serotypes of DENV, as smaller protein bands were noted in western blotting with anti-MFN1 and anti-MFN2 antibodies in cells infected with either one of the four DENV serotypes (Fig 6B). To examine whether expression of DENV protease alone is sufficient to cleave the endogenous MFNs, we checked the protein pattern of MFNs in cells with WT or protease-dead DENV protease whose IFN response was impaired in a protease activity dependent manner (S4 Fig) as reported in our previous study [35]. Smaller MFNs protein bands were detected in cells with WT but not the protease-dead S135A mutant of DENV NS2B3, especially in the presence of MG132 (Fig 6C).

**Fig 7:**
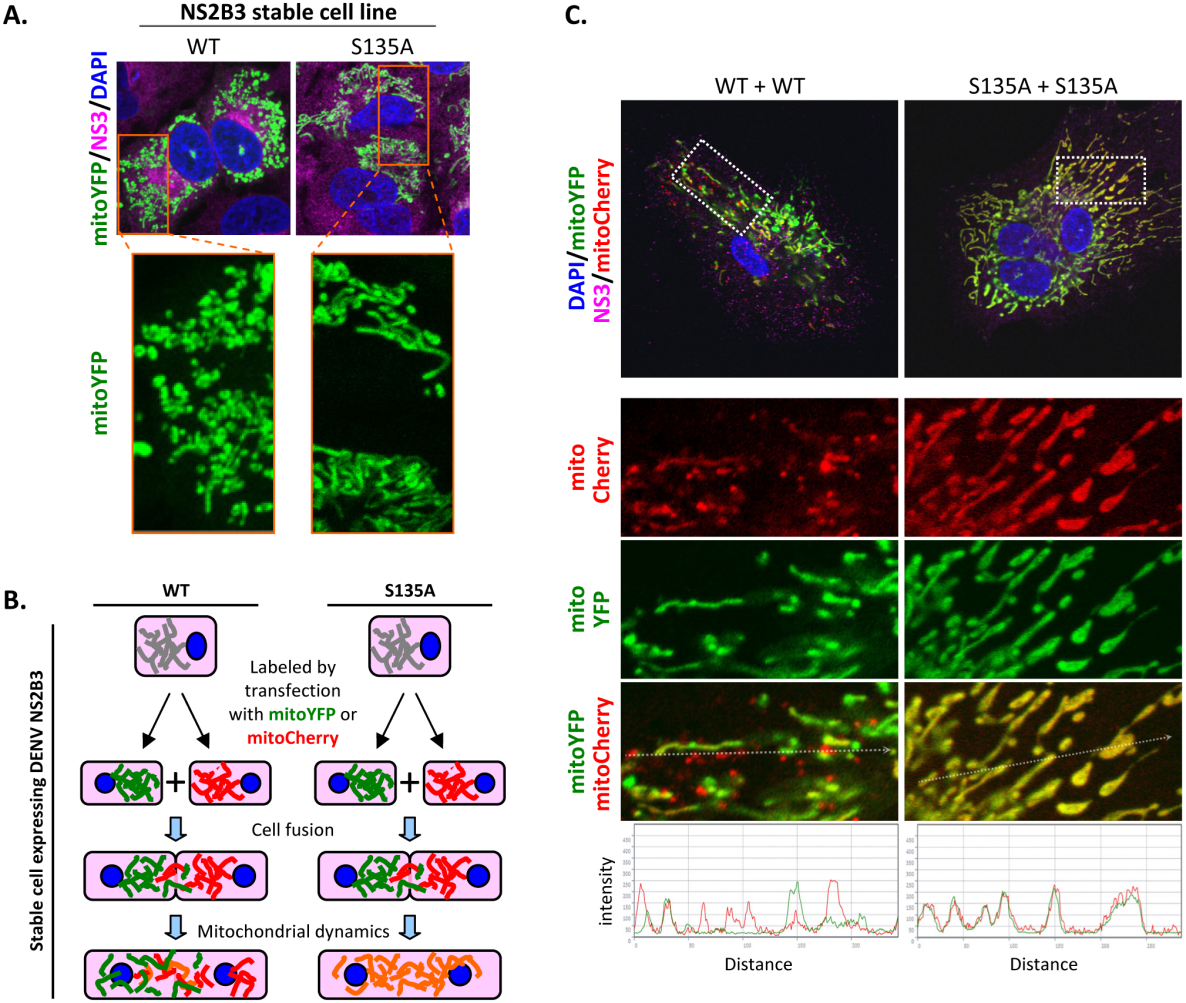
DENV protease governs mitochondrial morphology. (A) Confocal microscopy of A549 stable cells overexpressing DENV NS2B3 wild type (WT) or protease-dead mutant (S135A). Mitochondria were labeled by stably expressing mitoYFP and the magnified area is marked by a rectangle. Green: mitoYFP; magenta: DENV NS3; blue: DAPI. (B and C) Diagram (B) and the results (C) of mitochondria intermixing assay in cell hybrid harboring WT or mutated DENV protease. Mitochondria of A549 cells stably expressing WT or S135A-mutated DENV protease were labeled by transfection with mitoYFP or mitoCherry. Cells were fused by HVJ-E-mediated cell fusion, and the cell hybrids with or without DENV protease activity were magnified and analyzed for green and red fluorescence intensity by use of ZEN lite 2011 (Carl Zeiss MicroImaging GmbH). Green: mitoYFP; red: mitoCherry; magenta: DENV NS3.

Since only a small portion of MFNs was cleaved, we further studied whether this cleavage caused mitochondrial morphology change. Short/fragmented and tubular mitochondria were noted in cells stably expressing WT and protease-dead NS2B3, respectively (Fig 7A). Furthermore, mitochondrial dynamics measured by the intermixing experiment of A549 cells stably expressing WT or S135A-mutated NS2B3 labeled with mitoYFP or mitoCherry (outlined in Fig 7B) was hampered in cells with DENV protease. The signals of mitoYFP and mitoCherry were less colocalized in cell hybrid harboring wild type NS2B3, while even redistribution of these fluorescent signals were found in the hybrid with mutated NS2B3 (Fig 7C). Thus, even though the endogenous MFNs were not completely cleaved by NS2B3, DENV protease affects mitochondrial morphology by governing its dynamics.

### Manipulating MFNs expression governs consequences of DENV infection

**Fig 8:**
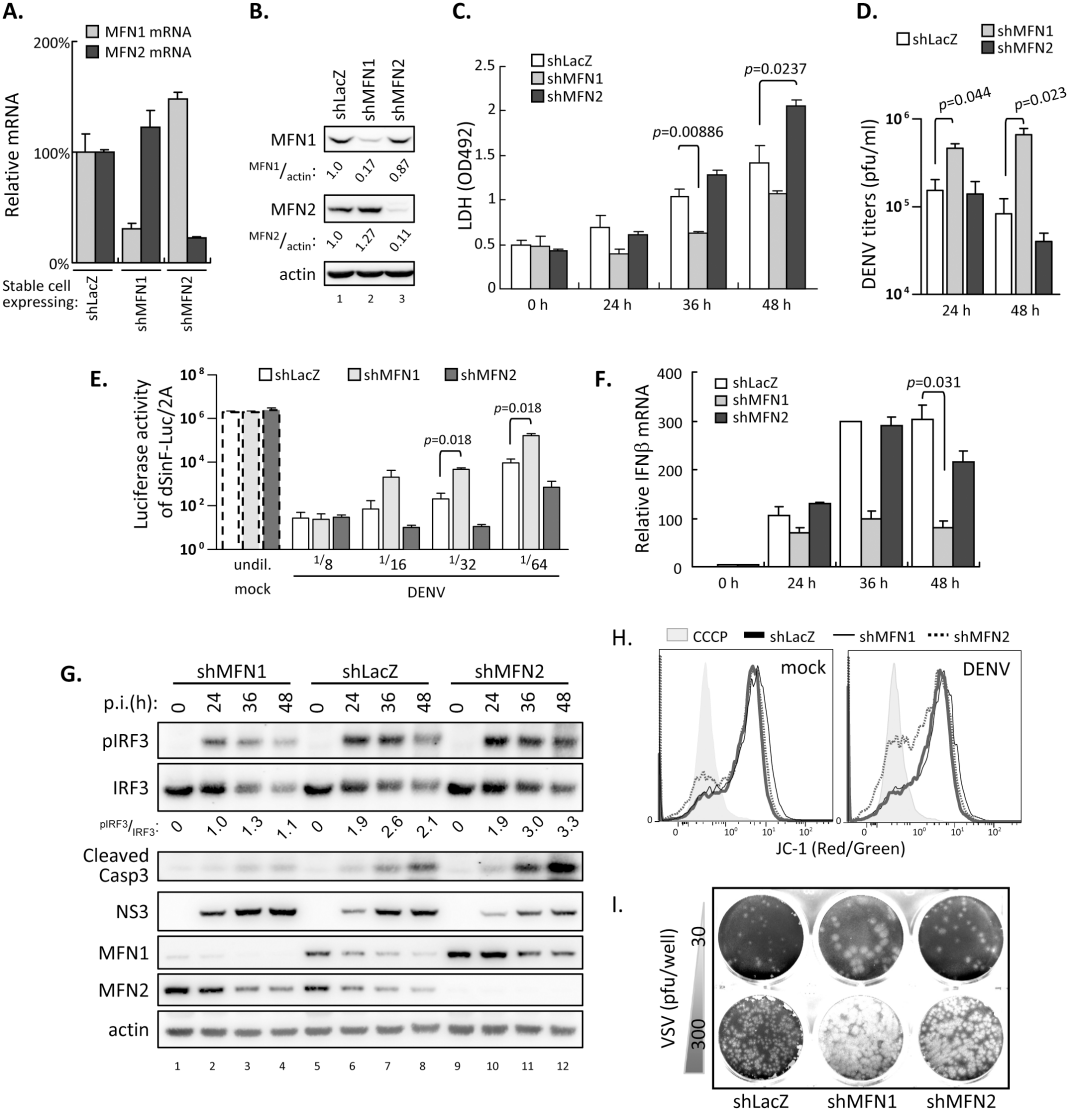
Silencing MFN1 or MFN2 reveals their distinct roles in DENV infection. (A and B) RT-qPCR (A) and western blot (B) validation of A549 cells stably expressing shRNA targeting LacZ, MFN1 or MFN2. Data in panels A and C-F are mean ± SD (*n = 3* per group) and were compared by two-tailed Student’s *t* test. Quantification in panel B is the relative ratio of the indicated protein to actin expression. (C) LDH release assay of shLacZ-, shMFN1- or shMFN2-expressing A549 cells infected with DENV serotype 2 (moi 10) for the indicated time. (D) DENV plaque-forming assay of shLacZ-, shMFN1- or shMFN2-expressing A549 cells infected with DENV (moi 0.1) for 24 or 48 h. (E) Analysis of antiviral activity in culture media from indicated cells with DENV infection (moi 5, 48 h) against dSinF-Luc/2A virus. (F) RT-qPCR analysis of IFNβ mRNA level in DENV infected (moi 10) A549-shLacZ, -shMFN1, and -shMFN2 cells for the indicated time. (G) Western blot analysis of DENV infected (moi 10) A549 cells expressing shLacZ, shMFN1 or shMFN2. The relative ratios of band intensity were quantified as indicated. (H) Flow cytometry with JC-1 staining of shLacZ-, shMFN1 or shMFN2-expressing A549 cells infected with DENV (moi 5) for 30 h. Decreased red/green fluorescence ratio of JC-1 represents disrupted MMP. CCCP, carbonyl cyanide *m*-chlorophenyl hydrazone, an ionophore that disrupts MMP. (I) Plaque forming assay of vesicular stomatitis virus (VSV) by using A549 cells expressing shLacZ, shMFN1 or shMFN2 as indicated. pfu, plaque-forming unit.

To validate the role of endogenous MFNs in DENV infection, we knocked down the expression of MFN1 or MFN2 in A549 cells by transduction with a lentivirus expressing short hairpin RNA (shRNA) targeting MFN1 or MFN2. The resulting A549-shMFN1 and -shMFN2 cells showed reduced mRNA and protein expression of MFN1 and 2, respectively (Fig 8A and 8B). A549 cells with MFN1 and MFN2 double knockdown harbored severe fragmented mitochondria and showed constitutive death, and cannot be included in this study. DENV-induced LDH release was attenuated in A549-shMFN1 cells but enhanced in cells with shMFN2 (Fig 8C). DENV viral production measured by plaque-forming assay was higher in A549-shMFN1 cells, whereas A549-shMFN2 cells produced similar levels of DENV as the shLacZ control (Fig 8D). Both of the antiviral activity against dSinF-Luc/2A in culture supernatants and IFNβ mRNA in cell lysates were reduced in samples from DENV-infected A549-shMFN1 cells, whereas those with MFN2 knockdown showed similar levels as the shLacZ control (Fig 8E and 8F). Consistently, lower level of pIRF3 was noted in cells with MFN1 knockdown (Fig 8G). Although MFN2 did not affect RLR signaling, knockdown of MFN2 deteriorated DENV-induced cytopathic effects measured by LDH release (Fig 8C), caspase 3 activation (Fig 8G), and MMP disruption in DENV-infected A549-shMFN2 cells (Fig 8H). Furthermore, vesicular stomatitis virus (VSV), an IFN-sensitive virus, formed enlarged plaques on A549-shMFN1 cells, and to a lesser extent on MFN2-knockdown cells, as compared to the shLacZ control (Fig 8I). Thus, MFN proteins participate in host defense against virus infection probably through promoting antiviral response and/or cell survival.

### Different functions played by MFN1 and MFN2 in MAVS signaling

**Fig 9:**
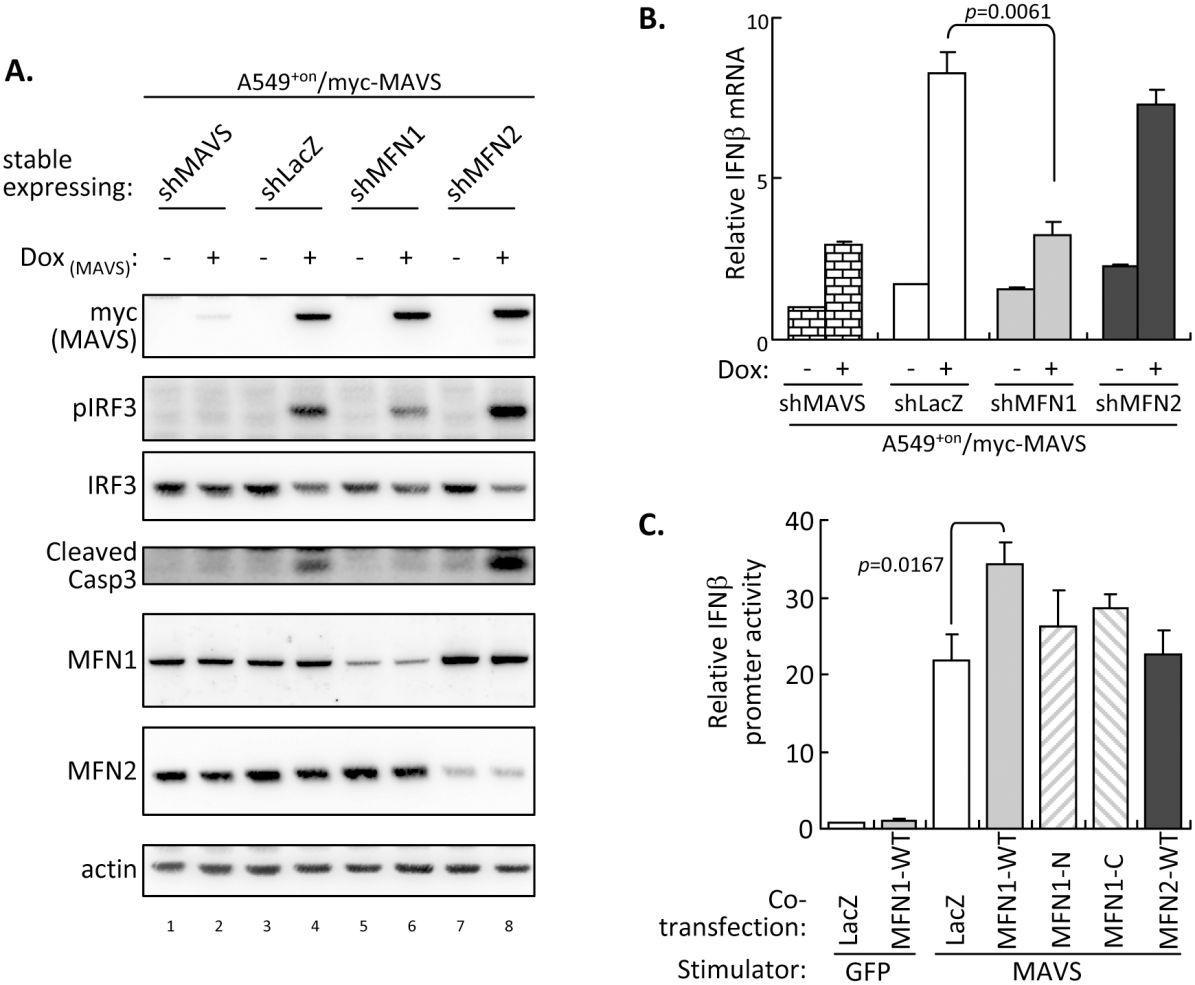
MFN1 and MFN2 manipulate MAVS-triggered signaling differently. (A and B) Western blot (A) and RT-qPCR (B) analysis of A549^+on^/myc-MAVS cells with stably expressing shRNA targeting the indicated genes after 48 h of Dox treatment. RT-qPCR data are mean ± SD (*n = 3* per group) and were compared by two-tailed Student’s *t* test. (C) Dual-luciferase activity analysis of A549 cells cotransfected with the indicated MFNs (cloned in pcDNA3.1 vector; 0.5 μg), stimulator or control (Flag-MAVS/pcDNA3 or Flag-GFP/pcDNA3; 0.4 μg), IRF3/pCR3.1 (0.3 μg), p125-Luc (0.2 μg) and pRL-TK (0.1 μg) for 24 h. The *firefly* luciferase activity (p125-Luc) was normalized to that of *renilla* luciferase (pRL-TK) and the relative luciferase activities are shown. Data are mean ± SD (*n = 3* per group) and were compared by two-tailed Student’s *t* test.

To clarify the role of MFN1 and MFN2 in MAVS signaling, we established A549^+on^/myc-MAVS inducible cell line with MFN1 or MFN2 knockdown. MAVS overexpression induced by Dox treatment triggered IRF3 phosphorylation, IFNβ induction and caspase 3 activation in cells with shLacZ control (Fig 9A, lane 4; and Fig 9B); however, different responses were noted in cells with shMFN1 or shMFN2 expression. Silencing MFN1 attenuated MAVS-triggered pIRF3, IFNβ induction and caspase 3 activation (Fig 9A, lane 6; and Fig 9B), but enhanced caspase 3 activation was seen in cells with reduced MFN2 (Fig 9A, lane 8). Furthermore, MFN1 alone cannot turn on IFNβ promoter but can enhance MAVS-triggered IFNβ promoter activation. However, MFN2 and the N-terminal or C-terminal cleaved products of MFN1 failed to facilitate MAVS-mediated IFNβ promoter activation (Fig 9C). Thus, MFN1 and MFN2 interplay with the antiviral IFN and cell death pathways in RLR-MAVS signaling differently. MFN1 can facilitate MAVS-mediated IFN production and caspase activation, whereas MFN2 is involved in blocking caspase activation. These data might help to explain the reason why DENV-induced cell death was attenuated in cells with either MFN1 overexpression or knockdown (Figs 3 and 8): MFN1 overexpression can protect cells from DENV infection by enhancing IFN response, while MFN1 knockdown can reduce the MAVS-mediated apoptosis leading to overall attenuation of cell death in DENV-infected cells.

### Cells with dominant-negative MFN1 show reduced RLR signaling and enhanced DENV infection

**Fig 10:**
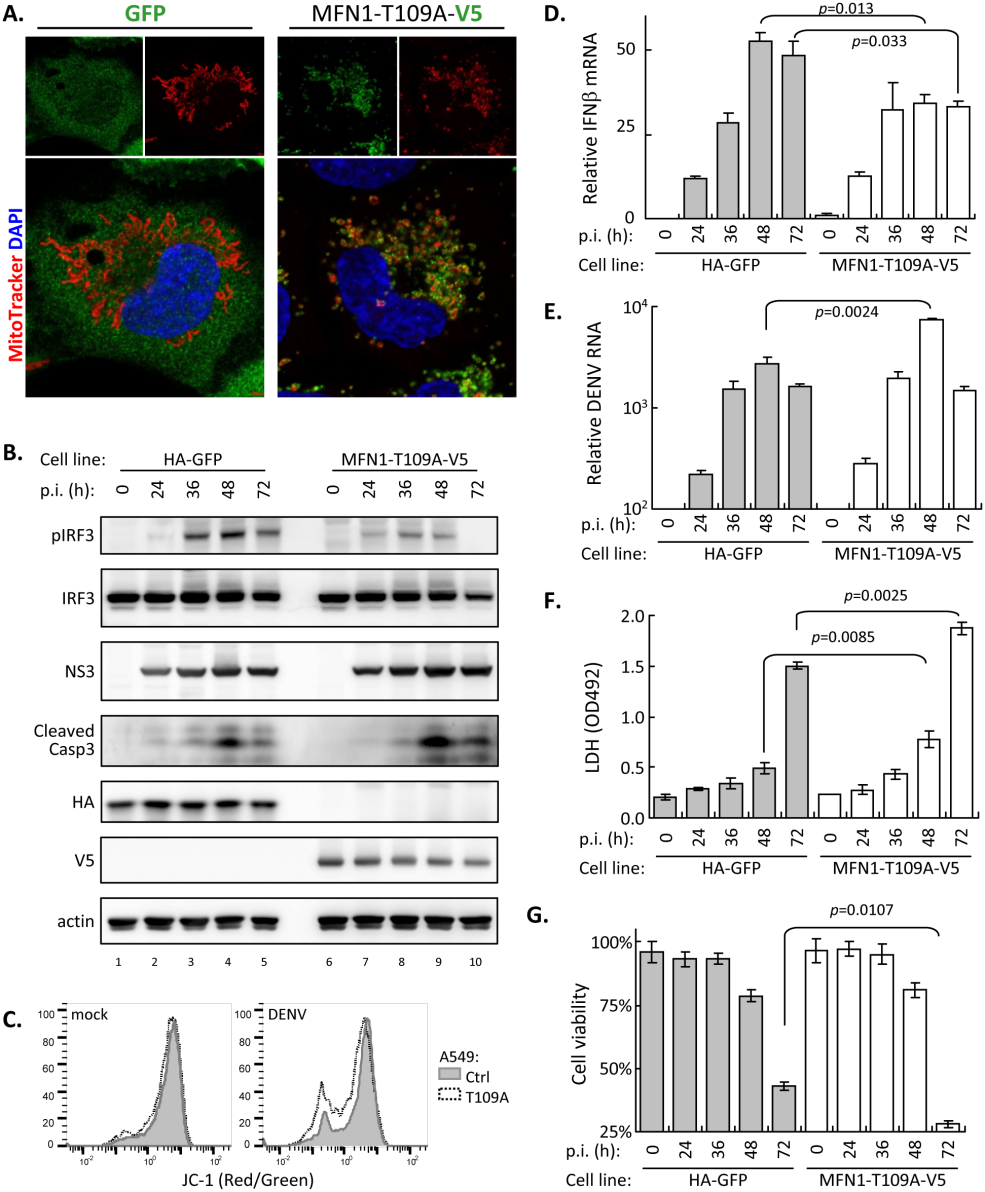
Blocking mitochondrial fusion attenuates RLR signaling and facilitates DENV infection. (A) Mitochondrial morphology analysis of A549 stable cell line overexpressing control GFP or V5-tagged MFN1-T109A. Morphology and subcellular localization of mitochondria and MFN1-T109A were revealed by MitoTracker and anti-V5 staining, respectively. (B) Western blot analysis of GFP- and MFN1-T109A-expressing A549 cells upon DENV infection. Cells were infected with DENV serotype 2 (moi 10) and harvested at indicated time. (C) MMP measurement of vector control (Ctrl) or MFN1-T109A-expressing cells after DENV infection. Cells were infected with DENV (moi 5) for 36 h and harvested for flow cytometry analysis with JC-1 staining. (D to G) Time course study of DENV infection in GFP- and MFN1-T109A-A549 cells. Samples were harvested for the quantification of intracellular IFNβ mRNA (D) and DENV RNA (E) by RT-qPCR, and for the measurement of LDH release (F) and cell viability by trypan blue exclusion (G). Data are mean ± SD (*n = 3* per group) and were compared by two-tailed Student’s *t* test.

To further address the importance of MFN1 in DENV infection, we established a stable cell line overexpressing a mutated MFN1-T109A [42], which dominant-negatively blocked mitochondrial fusion and resulted in fragmented mitochondria (Fig 10A). Compared to the GFP control cells, DENV-infected MFN1-T109A cells showed lower pIRF3, higher caspase 3 activation (Fig 10B) and more MMP disruption (Fig 10C). Consistently, MFN1-T109A cells induced lower IFNβ mRNA (Fig 10D), and resulted in higher DENV RNA replication (Fig 10E) and severer cytotoxicity measured by LDH release and cell viability (Fig 10F and 10G). Thus, mitochondria fusion contributes to host innate defense against DENV infection.

## Discussion

**Fig 11:**
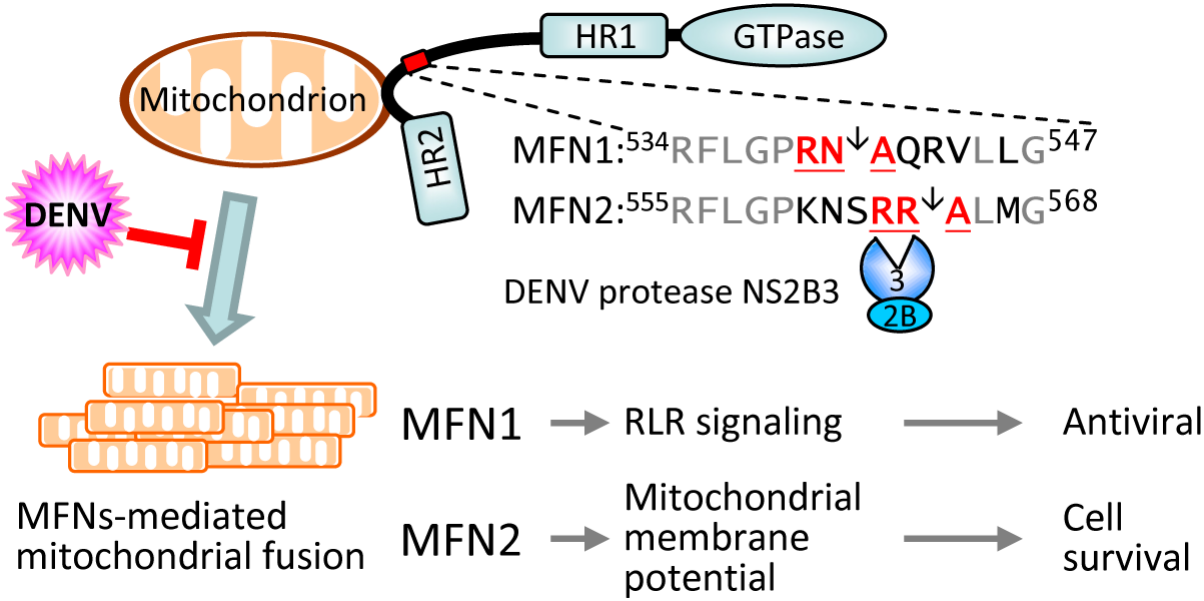
DENV governs MFN-mediated signaling by protein cleavage. MFN1 and MFN2 are involved in the initiation step of mitochondrial fusion by tethering mitochondria together. DENV infection suppresses mitochondrial fusion and mitochondrial dynamics by cleaving MFNs via viral protease NS2B3. Because MFN1 enhances host antiviral signaling and MFN2 maintains mitochondrial membrane potential during DENV infection, the cleavage of both MFNs by DENV protease would attenuate interferon production and increases cell death of DENV-infected cells.

Viruses depend on host cells to replicate and cellular factors involved in viral replication might be modulated during infection; these dysregulated cellular proteins/pathways may then contribute to viral pathogenesis. Various strategies have been adapted by viruses to modulate cellular proteins, for example DENV NS2B3, a protease essential for viral polyprotein processing, can cleave the IFN adaptor protein MITA/STING to subvert innate immunity [35,36]. Here, we identified two new cellular targets of DENV protease, MFN1 and MFN2, and also reveal their involvements in efficient RLR signaling and maintaining MMP in DENV-infected cells (as outlined in Fig 11). Downregulation of MFNs can impair mitochondrial dynamics and IFN signaling, thus promoting DENV replication and inducing cell death. Thus, to counteract the cellular antiviral responses, DENV protease not only cleaves IFN adaptor MITA/STING but also targets MFNs to blunt the mitochondria-mediated host defense.

Viruses affect mitochondrial dynamics, but the understanding of detailed mechanisms are limited to only a few cases [39,40,49]. Infection of alphaherpesviruses, such as herpes simplex virus type 1 and pseudorabies virus, blocks mitochondrial motility by reducing the recruitment of kinesin-1 to mitochondria via a viral glycoprotein B-dependent event [49]. Both HBV and HCV infection can promote mitochondrial fission by inducing the expression, phosphorylation and mitochondrial translocation of Drp1 [39,40]. Given that the dynamics and morphology of mitochondria are critical in innate immune response [20,22,23], the demonstration of particular viruses can manipulate mitochondrial dynamics has just begun to elucidate. Previously, DENV infection is known to cause apoptosis with mitochondrial involvement and DENV M protein has been shown to disrupt mitochondrial membrane potential [50,51]. Here, we provide the first example of viral modulating of mitochondria dynamics by cleaving MFNs and open a new research avenue on the interplay of viruses and cellular mitochondria.

Even though these two MFNs are similar in protein structure and function, compared with MFN1, MFN2 has lower GTPase activity [52] but has other specific functions such as oxidative metabolism [53,54], cell death involvement [55,56] and ER location in addition to mitochondria [19]. Previous findings on the roles of MFN1 and MFN2 on antiviral signaling are also not unified: MFN2, but not MFN1, interacts with MAVS and plays a suppressive role on the MAVS-triggered IFNβ promoter activation [24]; whereas subsequent reports showed that MFN1 interacts with MAVS and mitochondrial fusion is required for efficient RLR signaling [25,26]. Our findings that MFN1 and MFN2 play distinct roles in DENV infection, RLR signaling for MFN1 and MMP maintaining for MFN2, support that these two similar proteins are not functionally redundant in viral infection. MFNs may trigger mitochondria fusion to provide platforms for molecules in RLR signaling and anti-apoptotic pathways to interact with each other. However, it is not clear why MFN1 and MFN2 play different roles in DENV infection; possibilities, such as stronger GTPase activity of MFN1 and ER location of MFN2, might contribute to these phenomena and are awaited to be tested. Furthermore, DENV protease could cleave MFN1 and 2 at a region between the HR1 and TM domains, thus separating the GTPase domain from the HR2 domain and abolishing the proper function of MFNs. Our findings that DENV protease can cleave these two MFNs reinforce the importance of MFN-mediated cellular events in host defense against viral infection. The specific mechanisms contributed by MFN1/2 and the roles of these two MFNs in other viral infections remain to be further studied.

Malfunction of MFNs may cause severe diseases [15]. For example, mutations in the human *Mfn2* gene are responsible for the inherited disorder Charcot-Marie-Tooth neuropathy type 2A [57]. Downregulation of MFN protein is a common mechanism modulating the function of MFNs, in that ubiquitination of MFNs has been found in several organisms such as mammals, yeast and fly [58–60]. Parkin, an E3 ubiquitin ligase involved in mitochondrial integrity, induces the ubiquitination of MFN1 and 2, which leads to their degradation and prevents or delays mitochondria fusion [61]. JNK phosphorylation of MFN2 leads to the recruitment of the large HECT-domain E3 ubiquitin ligase Huwe1 and results in ubiqitination-mediated proteasomal degradation of MFN2 [62]. The ubiquitin-proteasome system also participates in downregulating MFNs in DENV-infected cells because the proteasome inhibitor MG132 could block the subsequent degradation and increase the visibility of the cleavage protein bands. Thus, the protease-cleaved MFNs fragments were further targeted by proteasome-mediated degradation. Murine MFN1, whose sequences at the putative DENV protease cleavage site are similar to that of human MFN1-ST mutant, was also slightly downregulated in DENV-infected murine PBMC, supporting the involvements of cellular protein degradation pathways. Moreover, only a portion of MFN1 and 2 was targeted by DENV protease, which suggests that specific subcellular location and/or certain stimuli are required for the cleavage of MFNs by DENV.

Mitochondria constantly undergo fusion and fission, and this dynamic behavior is critical for maintaining the structure and function of healthy mitochondria. The shift in mitochondrial dynamics toward fission and mitophagy to attenuate apoptosis may facilitate persistent HBV and HCV infection [39,40]. Cleavage of MFN1 and 2 during DENV infection may also contribute to the pathogenesis of DENV-related diseases. Our findings that DENV cleaves MFNs to modulate mitochondria-related events such as antiviral RLR signaling and induction of apoptosis open up new research directions on the interplay of DENV with various cellular functions related to mitochondrial dynamics.

## Materials and Methods

### Plasmids

The cDNAs of human *MFN1* and *MFN2* were cloned by PCR with the primers for BamH1-MFN1(-3~22) (5'-cgc*ggatcc*ataatggcagaacctgtttctccac-3') and Sac2-no stop-MFN1(2223–2200) (5'-atcc*ccgcgg*ggattcttcattgcttgaaggtag-3') for *MFN1*; and BamH1-MFN2(-3~20) (5'-cgc*ggatcc*gcaatgtccctgctcttctctcg-3') and Sac2-no stop-MFN2(2271–2249) (5'-atcc*ccgcgg*tctgctgggctgcaggtactggt-3') for *MFN2* (restriction enzyme recognition sites underlined). The sequences of cDNAs were the same as that for human *MFN1* (NM_033540) and *MFN2* (NM_001127660). The N-terminal HA-tag and C-terminal V5-tag were added as described [35]. Mutations of MFNs were introduced by single-primer PCR mutagenesis [63] with the primer sequences 5'-cttgtacatcgatttttgggcagtactaatgctcaaagggtgctcct-3' for MFN1-ST, 5'-tcgatttttgggccctagaagagctcaaagggtgctcctagga-3' for MFN1-NR, 5'-cgatttttgggccctagaaattgacatatggctcaaagggtgctcctagg-3' for MFN1-N, 5'-cctagtgggattggccatatagctaattgcttcctaagtgttgaagg-3' for MFN1-T109A, and 5'-cccaagaacagccgtcgagtactgatgggctacaatgaccagg-3' for MFN2-AV (mutation sequences underlined). The myc-tagged MFN1-C were cloned by PCR with the primers XhoI-MFN1(1621–1640): 5'-accg*ctcgag*gctcaaagggtgctcctagg-3' and p-MFN1(2226–2200)-stop: 5'-aaac*tta*ggattcttcattgcttgaaggtag-3'. The tag of Flag-MAVS [10] were replaced with myc tag in this study. The cDNAs for MFN1 and 2 were subcloned into pAS4w.1.Ppuro vector for lentivirus production. Lentiviral vectors with shRNAs targeting human MFN1 (5′-gctcaaagttgtaaatgcttt-3′, TRCN0000051837), human MFN2 (5′-gcaggtttactgcgaggaaat-3′, TRCN0000082684) or LacZ (5′-cgcgatcgtaatcacccgagt-3′, TRCN0000072224), and lentiviral vectors for the inducible expression system, pLAS_AS3w.aOn.Pbsd and pAS4w.1.Ppuro, were from the National RNAi Core Facility, Taiwan. Lentiviral vector pTY-EFeGFP [64] was used to stably express MFN1-T109A by replacing the GFP with V5-tagged MFN1-T109A. Preparation of lentivirus followed the protocol provided by the National RNAi Core Facility, Taiwan. The plasmid expressing mitochondrial RFP, mitoCherry, was cloned by replacing EYFP of pEYFP-mito (Clontech) with mCherry. The pCR3.1 vectors expressing NS2B3 and the S135A mutant of DENV and JEV were described previously [64].

### Viruses, cells, and chemicals

Propagation and titration of DENV-1 Hawaii, DENV-2 PL046, New Guinea C-N (NGC-N), DENV-3 739079A and DENV-4 866146A were as described [65]. Except indicated in Figs 1J and 6B, DENV-2 PL046 was used throughout this study. The IFN-sensitive sindbis reporter virus dSinF-Luc/2A was amplified and titrated in Vero cells (ATCC CCL-81) as described [35,43]. A549 cells (ATCC, CCL-185), a human lung carcinoma cell line, were cultured in F-12 medium supplemented with 10% fetal bovine serum (FBS). African green-monkey kidney Vero cells were cultured in MEM with 10% FBS and 2 mM L-glutamine. A549^+on^ was established by stable transduction of A549 cells with lentivirus generated from pLAS_AS3w.aOn.Pbsd plus the helper plasmids pCMVΔR8.91 and pMD.G (National RNAi Core Facility, Taiwan). Stable A549 cells overexpressing wild-type (WT) or protease-dead mutant (S135A) of DENV NS2B3 were previously described [35]. MitoTracker Orange CMTMRos (Molecular Probes) and Lipofectamine 2000 (Invitrogen) were used.

### Western blot analysis

Protein samples were prepared by using RIPA lysis buffer (10 mM Tris, pH 7.5, 5 mM EDTA, 150 mM NaCl, 0.1% SDS, 1% TritonX-100, 1% sodium deoxycholate) containing protease and phosphatase inhibitors (Roche). After SDS-PAGE separation, samples were transferred to a nitrocellulose membrane (Hybond-C Super, Amersham), which was blocked with skim milk in phosphate buffered saline (PBS) with 0.1% Tween-20 (PBS-T), then reacted with the primary antibodies for caspase-3 (#9662) and phospho-IRF3 (S396) (#4961; both Cell Signaling); IRF3 (sc-9082), MFN1 (sc-50330), and MFN2 (sc-100560; all Santa Cruz Biotechnology); OPA1 (GTX48589), and Fis1 (GTX111010; both GeneTex); actin (NB600-501; Novus Biologicals); HA (#3724), myc (#2278; both Cell Signaling); V5 (R960-25; Life Technologies); and Flag M2 (F1804; Sigma-Aldrich). Blots were treated with horseradish peroxidase-conjugated secondary antibody (111-035-144 and 115-035-146; Jackson ImmunoResearch), and signals were detected by enhanced chemiluminescence (Pierce). The relative ratios of band density were quantified by ImageJ.

### Coimmunoprecipitation

Cells were lysed with protein lysis buffer containing a cocktail of protease and phosphatase inhibitors (Roche). Cell lysates were immunoprecipitated with mouse anti-Flag IgG beads, control IgG beads (both Sigma-Aldrich) or rabbit anti-NS3 antibody (1:250; GTX124252; GeneTex) for overnight at 4°C. The immunocomplex was captured by use of Protein G Mag Sepharose (GE Healthcare) at 4°C for 1 h, washed with protein lysis buffer, resuspended in sample buffer with 2-mercaptoethanol, and then examined by Western blot analysis.

### RT-qPCR analysis

Total RNA was prepared with an RNeasy RNA Mini Kit (Qiagen) and the cDNA was reverse transcribed from 1 μg of total RNA with random hexamer primer using a ThermoScript RT kit (Invitrogen). qPCR was then carried out using the specific primer sets for DENV RNA (5'-tatccaatgcctctgggaac-3' and 5'-tggctcgtaagtggctttct-3'), MFN1 (5'-cataatggcagaacctgtttctccac-3' and 5'-gctttatctccatcagttccttcaacactt-3'), MFN2 (5'-gcaatgtccctgctcttctctcg-3' and 5'-ctcactgatgcctctcactttggatag-3'), IFNβ (5'-cacgacagctctttccatga-3' and 5'-agccagtgctcgatgaatct-3') and actin (5'-tcctgtggcatccacgaaact-3' and 5'-gaagcatttgcggtggacgat-3') with the LightCycler FastStart DNA Master PLUS SYBR Green I kit (Roche), according to the manufacturer’s recommendations. The level of each gene expression was normalized to that of actin based on the second derivative maximum method (Roche). Melting curves were used to verify the specificity of PCR products.

### Cytotoxicity assay

Trypan blue exclusion test was performed for cell viability. The release of the cytoplasmic enzyme lactate dehydrogenase (LDH) was measured using a cytotoxicity detection kit (Roche).

### Antiviral activity assay

As previously described [35], conditioned medium was UV-inactivated and then serial-diluted with fresh medium. Vero cells were cultured with the conditioned medium for 18 h, then infected with dSinF-Luc/2A virus for 24 h. Cell lysates were harvested for luciferase activity assay (Promega).

### Flow cytometry

For MMP and annexin V analyses, cells were detached and resuspended in 1 ml warm medium. After 15 to 30 min of JC-1 (1 μM) labeling, cells were stained with annexin V-Cy5 (BioVision), then analyzed by use of the FACSCanto flow cytometer and FACSDiva software (Becton Dickinson) as described [10].

### Fluorescence microscopy

Cells were incubated with MitoTracker (1 nM) for 30 min in prewarmed complete medium, fixed with prewarmed 4% paraformaldehyde in PBS for 10 min to preserve MitoTracker signal, and then permeabilized by 0.5% TritonX-100 in the dark. After being blocked with skim milk in PBS-T, antibodies against HA-tag (Cell Signaling) and NS3 [35] were added for overnight. Alexa Fluor-488-conjugated secondary antibody (Molecular Probes) was added for 1 h at room temperature, and cell nuclei were counterstained with DAPI. For samples examined by confocal laser-scanning microscopy (LSM 510, Zeiss), cells were seeded in μ-Slides chamber slides (ibidi) overnight before treatments. For live confocal microscopy, samples were examined by use of the UltraVIEW VoX live cell imaging system with Volocity software (PerkinElmer). For high-content analysis of mitochondrial morphology, we used the ImageXpress Micro Image XL System (Molecular Devices).

### Mitochondrial dynamics assays

For the mitochondria intermixing experiment, cell fusion was done by using HVJ Envelope Cell Fusion Kit (Cosmo Bio) with previously described modifications [38]. Briefly, A549/mitoYFP cells were detached and treated with HVJ-E at 4°C for 5 min, then overlaid onto semi-confluent A549/mitoCherry cells. HVJ-E-mediated cell membrane fusion was initiated in the presence of translation inhibitor cyclohexamide (50 μg/ml) by increasing the temperature to 37°C. For DENV-infection group, A549/mitoYFP and A549/mitoCherry cells were infected with DENV (moi 10) for 24 h, then HVJ-E fusion kit was used as the mock-infection group.

### Isolation of DENV-infected murine peripheral blood mononuclear cells (PBMCs)

STAT1^-/-^ mice [66] were challenged intraperitoneally with 1x10^6^ plaque-forming units of DENV (serotype 2, mouse-adapted neurovirulent strain NGC-N) in 500 μl of PBS and simultaneously injected intracranially with 30 μl of PBS into the right hemisphere of mouse brains [67]. PBMCs were isolated from the whole blood by standard density-gradient centrifugation with Ficoll-Paque (Amersham Biosciences).

### Ethics statement

Animal studies were conducted according to the guidelines outlined by Council of Agriculture, Executive Yuan, Republic of China. The animal protocol was approved by the Academia Sinica Institutional Animal Care and Utilization Committee (Protocol ID 11-11-245). All surgery was performed under sodium pentobarbital anesthesia and every effort was made to minimize suffering.

### Statistical analysis

Data are presented as mean ± SD and were compared by two-tailed Student’s *t* test, or analyzed by one way ANOVA and Bonferroni multiple-comparison test as mentioned in figure legends.

## Supporting Information

S1 Movie Real-time observation of mitochondria movement. A549 cells were infected with DENV serotype 2 for 24 h and stained with MitoTracker for 30 min. The mitochondrial dynamics of mock- and DENV-infected cells were monitored in real-time photography. A random selected field of each condition was shown as animation by playing photos at 30 fps (frames per second).
(AVI)

S2 Movie DENV infection suppresses MFN1-triggered mitochondrial fusion/aggregation. A549^+on^/HA-MFN1 cells were infected with DENV serotype 2 (moi 10) for 36 h and stained with MitoTracker before Dox induction. The mitochondrial dynamics of mock- (left) and DENV-infected (right) cells were photographed side by side every 30 sec continuously after Dox treatment. A representative cell of each condition is animated by playing photos at 30 fps.
(AVI)

S1 Fig Analysis of mitochondrial movement in DENV infected A549 cells. The live confocal microscopy of S1 Movie was composed of photos taken every 0.255 sec. Since the difference between two sequential images within identical field can be subtracted as the movement [61], the sequential mitochondrial movements of DENV-infected cells within 30 sec were analyzed frame-by-frame (117 frames) by Volocity and MetaMorph software and normalized to that of mock. The result is shown as mean ± SD (*n = 116*, per group) and present in percentage.
(TIF)

S2 Fig CCCP treatment triggers mitochondria fragmentation. (A and B) A549 cells stably expressing mitoCherry were treated with the ionophore carbonyl cyanide m-chlorophenyl hydrazone (CCCP; 100 μM) or not (mock) for 4 h and analyzed by fluorescent microscopy (A) and by immunoblotting (B). DENV, DENV-infected for 48 h (serotype 2, moi 10).
(TIF)

S3 Fig MFNs trigger mitochondrial hyperfusion in cells expressing protease-dead DENV NS2B3. Confocal microscopy of A549 cells cotransfected with Flag-tagged DENV NS2B3(S135A) and the indicated wild-type and mutated MFN constructs for 24 h. Arrows indicate the cells expressing both Flag-tagged DENV protease and HA-tagged MFN. Green: anti-HA; magenta: anti-Flag; red: MitoTracker; blue: DAPI.
(TIF)

S4 Fig DENV protease suppresses JEV-induced IFN induction signaling. (A and B) A549 cells stably expressing wild-type (WT) or protease-dead (S135A) DENV protease NS2B3 were infected with JEV (moi 10) for 24 h and analyzed by immunoblotting (A) and by RT-qPCR (B). RQ, relative quantification. Note that anti-NS3 antibody recognizes both JEV and DENV NS3.
(TIF)

## References

[1] Vandecasteele G, Szabadkai G, Rizzuto R. Mitochondrial calcium homeostasis: mechanisms and molecules. IUBMB Life. 2001;52(3–5):213–9. 11798035 10.1080/15216540152846028

[2] Wang C, Youle RJ. The role of mitochondria in apoptosis. Annu Rev Genet. 2009;43:95–118. 10.1146/annurev-genet-102108-134850 19659442 PMC4762029

[3] Galluzzi L, Kepp O, Kroemer G. Mitochondria: master regulators of danger signalling. Nat Rev Mol Cell Biol. 2012;13(12):780–8. 10.1038/nrm3479 23175281

[4] Tait SW, Green DR. Mitochondria and cell signalling. J Cell Sci. 2012;125(Pt 4):807–15. 10.1242/jcs.099234 22448037 PMC3311926

[5] Kawai T, Takahashi K, Sato S, Coban C, Kumar H, Kato H, et al IPS-1, an adaptor triggering RIG-I- and Mda5-mediated type I interferon induction. Nat Immunol. 2005;6(10):981–8. 16127453 10.1038/ni1243

[6] Meylan E, Curran J, Hofmann K, Moradpour D, Binder M, Bartenschlager R, et al Cardif is an adaptor protein in the RIG-I antiviral pathway and is targeted by hepatitis C virus. Nature. 2005;437(7062):1167–72. 16177806 10.1038/nature04193

[7] Seth RB, Sun L, Ea CK, Chen ZJ. Identification and characterization of MAVS, a mitochondrial antiviral signaling protein that activates NF-kappaB and IRF 3. Cell. 2005;122(5):669–82. 16125763 10.1016/j.cell.2005.08.012

[8] Xu LG, Wang YY, Han KJ, Li LY, Zhai Z, Shu HB. VISA is an adapter protein required for virus-triggered IFN-beta signaling. Mol Cell. 2005;19(6):727–40. 16153868 10.1016/j.molcel.2005.08.014

[9] Lei Y, Moore CB, Liesman RM, O'Connor BP, Bergstralh DT, Chen ZJ, et al MAVS-mediated apoptosis and its inhibition by viral proteins. PLoS One. 2009;4(5):e5466 10.1371/journal.pone.0005466 19404494 PMC2674933

[10] Yu CY, Chiang RL, Chang TH, Liao CL, Lin YL. The interferon stimulator mitochondrial antiviral signaling protein facilitates cell death by disrupting the mitochondrial membrane potential and by activating caspases. J Virol. 2010;84(5):2421–31. 10.1128/JVI.02174-09 20032188 PMC2820939

[11] Palmer CS, Osellame LD, Stojanovski D, Ryan MT. The regulation of mitochondrial morphology: intricate mechanisms and dynamic machinery. Cell Signal. 2011;23(10):1534–45. 10.1016/j.cellsig.2011.05.021 21683788

[12] van der Bliek AM, Shen Q, Kawajiri S. Mechanisms of mitochondrial fission and fusion. Cold Spring Harb Perspect Biol. 2013;5(6):a011072 10.1101/cshperspect.a011072 23732471 PMC3660830

[13] Chan DC. Fusion and fission: interlinked processes critical for mitochondrial health. Annu Rev Genet. 2012;46:265–87. 10.1146/annurev-genet-110410-132529 22934639

[14] Youle RJ, van der Bliek AM. Mitochondrial fission, fusion, and stress. Science. 2012;337(6098):1062–5. 10.1126/science.1219855 22936770 PMC4762028

[15] Santel A. Get the balance right: mitofusins roles in health and disease. Biochim Biophys Acta. 2006;1763(5–6):490–9. 16574259 10.1016/j.bbamcr.2006.02.004

[16] Liesa M, Palacin M, Zorzano A. Mitochondrial dynamics in mammalian health and disease. Physiol Rev. 2009;89(3):799–845. 10.1152/physrev.00030.2008 19584314

[17] Koshiba T, Detmer SA, Kaiser JT, Chen H, McCaffery JM, Chan DC. Structural basis of mitochondrial tethering by mitofusin complexes. Science. 2004;305(5685):858–62. 15297672 10.1126/science.1099793

[18] Zorzano A, Liesa M, Sebastian D, Segales J, Palacin M. Mitochondrial fusion proteins: dual regulators of morphology and metabolism. Semin Cell Dev Biol. 2010;21(6):566–74. 10.1016/j.semcdb.2010.01.002 20079867

[19] de Brito OM, Scorrano L. Mitofusin 2 tethers endoplasmic reticulum to mitochondria. Nature. 2008;456(7222):605–10. 10.1038/nature07534 19052620

[20] Arnoult D, Soares F, Tattoli I, Girardin SE. Mitochondria in innate immunity. EMBO Rep. 2011;12(9):901–10. 10.1038/embor.2011.157 21799518 PMC3166463

[21] Koshiba T, Bashiruddin N, Kawabata S. Mitochondria and antiviral innate immunity. Int J Biochem Mol Biol. 2011;2(3):257–62. 22003438 PMC3193288

[22] West AP, Shadel GS, Ghosh S. Mitochondria in innate immune responses. Nat Rev Immunol. 2011;11(6):389–402. 10.1038/nri2975 21597473 PMC4281487

[23] Koshiba T, Yasukawa K, Yanagi Y, Kawabata S. Mitochondrial membrane potential is required for MAVS-mediated antiviral signaling. Sci Signal. 2011;4(158):ra7 10.1126/scisignal.2001147 21285412

[24] Yasukawa K, Oshiumi H, Takeda M, Ishihara N, Yanagi Y, Seya T, et al Mitofusin 2 inhibits mitochondrial antiviral signaling. Sci Signal. 2009;2(84):ra47 10.1126/scisignal.2000287 19690333

[25] Castanier C, Garcin D, Vazquez A, Arnoult D. Mitochondrial dynamics regulate the RIG-I-like receptor antiviral pathway. EMBO Rep. 2010;11(2):133–8. 10.1038/embor.2009.258 20019757 PMC2828750

[26] Onoguchi K, Onomoto K, Takamatsu S, Jogi M, Takemura A, Morimoto S, et al Virus-infection or 5'ppp-RNA activates antiviral signal through redistribution of IPS-1 mediated by MFN1. PLoS Pathog. 2010;6(7):e1001012 10.1371/journal.ppat.1001012 20661427 PMC2908619

[27] Simmons CP, Farrar JJ, Nguyen v V, Wills B. Dengue. N Engl J Med. 2012;366(15):1423–32. 10.1056/NEJMra1110265 22494122

[28] Bhatt S, Gething PW, Brady OJ, Messina JP, Farlow AW, Moyes CL, et al The global distribution and burden of dengue. Nature. 2013;496(7446):504–7. 10.1038/nature12060 23563266 PMC3651993

[29] Chang TH, Liao CL, Lin YL. Flavivirus induces interferon-beta gene expression through a pathway involving RIG-I-dependent IRF-3 and PI3K-dependent NF-kappaB activation. Microbes Infect. 2006;8(1):157–71. 16182584 10.1016/j.micinf.2005.06.014

[30] Loo YM, Fornek J, Crochet N, Bajwa G, Perwitasari O, Martinez-Sobrido L, et al Distinct RIG-I and MDA5 signaling by RNA viruses in innate immunity. J Virol. 2008;82(1):335–45. 17942531 10.1128/JVI.01080-07 PMC2224404

[31] Perry ST, Prestwood TR, Lada SM, Benedict CA, Shresta S. Cardif-mediated signaling controls the initial innate response to dengue virus in vivo. J Virol. 2009;83(16):8276–81. 10.1128/JVI.00365-09 19494017 PMC2715757

[32] Li XD, Sun L, Seth RB, Pineda G, Chen ZJ. Hepatitis C virus protease NS3/4A cleaves mitochondrial antiviral signaling protein off the mitochondria to evade innate immunity. Proc Natl Acad Sci U S A. 2005;102(49):17717–22. 16301520 10.1073/pnas.0508531102 PMC1308909

[33] Yang Y, Liang Y, Qu L, Chen Z, Yi M, Li K, et al Disruption of innate immunity due to mitochondrial targeting of a picornaviral protease precursor. Proc Natl Acad Sci U S A. 2007;104(17):7253–8. 17438296 10.1073/pnas.0611506104 PMC1855380

[34] Wang B, Xi X, Lei X, Zhang X, Cui S, Wang J, et al Enterovirus 71 protease 2Apro targets MAVS to inhibit anti-viral type I interferon responses. PLoS Pathog. 2013;9(3):e1003231 10.1371/journal.ppat.1003231 23555247 PMC3605153

[35] Yu CY, Chang TH, Liang JJ, Chiang RL, Lee YL, Liao CL, et al Dengue virus targets the adaptor protein MITA to subvert host innate immunity. PLoS Pathog. 2012;8(6):e1002780 10.1371/journal.ppat.1002780 22761576 PMC3386177

[36] Aguirre S, Maestre AM, Pagni S, Patel JR, Savage T, Gutman D, et al DENV inhibits type I IFN production in infected cells by cleaving human STING. PLoS Pathog. 2012;8(10):e1002934 10.1371/journal.ppat.1002934 23055924 PMC3464218

[37] Chen H, Detmer SA, Ewald AJ, Griffin EE, Fraser SE, Chan DC. Mitofusins Mfn1 and Mfn2 coordinately regulate mitochondrial fusion and are essential for embryonic development. J Cell Biol. 2003;160(2):189–200. 12527753 10.1083/jcb.200211046 PMC2172648

[38] Eura Y, Ishihara N, Yokota S, Mihara K. Two mitofusin proteins, mammalian homologues of FZO, with distinct functions are both required for mitochondrial fusion. J Biochem. 2003;134(3):333–44. 14561718 10.1093/jb/mvg150

[39] Kim SJ, Khan M, Quan J, Till A, Subramani S, Siddiqui A. Hepatitis B virus disrupts mitochondrial dynamics: induces fission and mitophagy to attenuate apoptosis. PLoS Pathog. 2013;9(12):e1003722 10.1371/journal.ppat.1003722 24339771 PMC3855539

[40] Kim SJ, Syed GH, Khan M, Chiu WW, Sohail MA, Gish RG, et al Hepatitis C virus triggers mitochondrial fission and attenuates apoptosis to promote viral persistence. Proc Natl Acad Sci U S A. 2014;111(17):6413–8. 10.1073/pnas.1321114111 24733894 PMC4035934

[41] Park KS, Wiederkehr A, Kirkpatrick C, Mattenberger Y, Martinou JC, Marchetti P, et al Selective actions of mitochondrial fission/fusion genes on metabolism-secretion coupling in insulin-releasing cells. J Biol Chem. 2008;283(48):33347–56. 10.1074/jbc.M806251200 18832378 PMC2662262

[42] Santel A, Frank S, Gaume B, Herrler M, Youle RJ, Fuller MT. Mitofusin-1 protein is a generally expressed mediator of mitochondrial fusion in mammalian cells. J Cell Sci. 2003;116(Pt 13):2763–74. 12759376 10.1242/jcs.00479

[43] Huang PY, Guo JH, Hwang LH. Oncolytic Sindbis virus targets tumors defective in the interferon response and induces significant bystander antitumor immunity in vivo. Mol Ther. 2012;20(2):298–305. 10.1038/mt.2011.245 22068428 PMC3277240

[44] Preugschat F, Yao CW, Strauss JH. In vitro processing of dengue virus type 2 nonstructural proteins NS2A, NS2B, and NS3. J Virol. 1990;64(9):4364–74. 2143543 10.1128/jvi.64.9.4364-4374.1990 PMC247904

[45] Wengler G, Czaya G, Farber PM, Hegemann JH. In vitro synthesis of West Nile virus proteins indicates that the amino-terminal segment of the NS3 protein contains the active centre of the protease which cleaves the viral polyprotein after multiple basic amino acids. J Gen Virol. 1991;72(Pt 4):851–8. 1826736 10.1099/0022-1317-72-4-851

[46] Zhang L, Mohan PM, Padmanabhan R. Processing and localization of Dengue virus type 2 polyprotein precursor NS3-NS4A-NS4B-NS5. J Virol. 1992;66(12):7549–54. 1433530 10.1128/jvi.66.12.7549-7554.1992 PMC240467

[47] Yusof R, Clum S, Wetzel M, Murthy HM, Padmanabhan R. Purified NS2B/NS3 serine protease of dengue virus type 2 exhibits cofactor NS2B dependence for cleavage of substrates with dibasic amino acids in vitro. J Biol Chem. 2000;275(14):9963–9. 10744671 10.1074/jbc.275.14.9963

[48] Bera AK, Kuhn RJ, Smith JL. Functional characterization of cis and trans activity of the Flavivirus NS2B-NS3 protease. J Biol Chem. 2007;282(17):12883–92. 17337448 10.1074/jbc.M611318200

[49] Kramer T, Enquist LW. Alphaherpesvirus infection disrupts mitochondrial transport in neurons. Cell Host Microbe. 2012;11(5):504–14. 10.1016/j.chom.2012.03.005 22607803 PMC3358700

[50] Catteau A, Kalinina O, Wagner MC, Deubel V, Courageot MP, Despres P. Dengue virus M protein contains a proapoptotic sequence referred to as ApoptoM. J Gen Virol. 2003;84(Pt 10):2781–93. 13679613 10.1099/vir.0.19163-0

[51] Brabant M, Baux L, Casimir R, Briand JP, Chaloin O, Porceddu M, et al A flavivirus protein M-derived peptide directly permeabilizes mitochondrial membranes, triggers cell death and reduces human tumor growth in nude mice. Apoptosis. 2009;14(10):1190–203. 10.1007/s10495-009-0394-y 19693674

[52] Ishihara N, Eura Y, Mihara K. Mitofusin 1 and 2 play distinct roles in mitochondrial fusion reactions via GTPase activity. J Cell Sci. 2004;117(Pt 26):6535–46. 15572413 10.1242/jcs.01565

[53] Bach D, Pich S, Soriano FX, Vega N, Baumgartner B, Oriola J, et al Mitofusin-2 determines mitochondrial network architecture and mitochondrial metabolism. A novel regulatory mechanism altered in obesity. J Biol Chem. 2003;278(19):17190–7. 12598526 10.1074/jbc.M212754200

[54] Pich S, Bach D, Briones P, Liesa M, Camps M, Testar X, et al The Charcot-Marie-Tooth type 2A gene product, Mfn2, up-regulates fuel oxidation through expression of OXPHOS system. Hum Mol Genet. 2005;14(11):1405–15. 15829499 10.1093/hmg/ddi149

[55] Parra V, Eisner V, Chiong M, Criollo A, Moraga F, Garcia A, et al Changes in mitochondrial dynamics during ceramide-induced cardiomyocyte early apoptosis. Cardiovasc Res. 2008;77(2):387–97. 18006463 10.1093/cvr/cvm029

[56] Ngoh GA, Papanicolaou KN, Walsh K. Loss of mitofusin 2 promotes endoplasmic reticulum stress. J Biol Chem. 2012;287(24):20321–32. 10.1074/jbc.M112.359174 22511781 PMC3370214

[57] Zuchner S, Mersiyanova IV, Muglia M, Bissar-Tadmouri N, Rochelle J, Dadali EL, et al Mutations in the mitochondrial GTPase mitofusin 2 cause Charcot-Marie-Tooth neuropathy type 2A. Nat Genet. 2004;36(5):449–51. 15064763 10.1038/ng1341

[58] Cohen MM, Leboucher GP, Livnat-Levanon N, Glickman MH, Weissman AM. Ubiquitin-proteasome-dependent degradation of a mitofusin, a critical regulator of mitochondrial fusion. Mol Biol Cell. 2008;19(6):2457–64. 10.1091/mbc.E08-02-0227 18353967 PMC2397313

[59] Ziviani E, Tao RN, Whitworth AJ. Drosophila parkin requires PINK1 for mitochondrial translocation and ubiquitinates mitofusin. Proc Natl Acad Sci U S A. 2010;107(11):5018–23. 10.1073/pnas.0913485107 20194754 PMC2841909

[60] Gegg ME, Cooper JM, Chau KY, Rojo M, Schapira AH, Taanman JW. Mitofusin 1 and mitofusin 2 are ubiquitinated in a PINK1/parkin-dependent manner upon induction of mitophagy. Hum Mol Genet. 2010;19(24):4861–70. 10.1093/hmg/ddq419 20871098 PMC3583518

[61] Tanaka A, Cleland MM, Xu S, Narendra DP, Suen DF, Karbowski M, et al Proteasome and p97 mediate mitophagy and degradation of mitofusins induced by Parkin. J Cell Biol. 2010;191(7):1367–80. 10.1083/jcb.201007013 21173115 PMC3010068

[62] Leboucher GP, Tsai YC, Yang M, Shaw KC, Zhou M, Veenstra TD, et al Stress-induced phosphorylation and proteasomal degradation of mitofusin 2 facilitates mitochondrial fragmentation and apoptosis. Mol Cell. 2012;47(4):547–57. 10.1016/j.molcel.2012.05.041 22748923 PMC3526191

[63] Makarova O, Kamberov E, Margolis B. Generation of deletion and point mutations with one primer in a single cloning step. Biotechniques. 2000;29(5):970–2. 11084856 10.2144/00295bm08

[64] Yu CY, Hsu YW, Liao CL, Lin YL. Flavivirus infection activates the XBP1 pathway of the unfolded protein response to cope with endoplasmic reticulum stress. J Virol. 2006;80(23):11868–80. 16987981 10.1128/JVI.00879-06 PMC1642612

[65] Lin YL, Liao CL, Chen LK, Yeh CT, Liu CI, Ma SH, et al Study of Dengue virus infection in SCID mice engrafted with human K562 cells. J Virol. 1998;72(12):9729–37. 9811707 10.1128/jvi.72.12.9729-9737.1998 PMC110483

[66] Durbin JE, Hackenmiller R, Simon MC, Levy DE. Targeted disruption of the mouse Stat1 gene results in compromised innate immunity to viral disease. Cell. 1996;84(3):443–50. 8608598 10.1016/s0092-8674(00)81289-1

[67] Chen ST, Lin YL, Huang MT, Wu MF, Cheng SC, Lei HY, et al CLEC5A is critical for dengue-virus-induced lethal disease. Nature. 2008;453(7195):672–6. 10.1038/nature07013 18496526

