# Supplementary figures and images for: Dengue Virus Impairs Mitochondrial Fusion by Cleaving Mitofusins
Source: PLoS Pathog. 2015 Dec 30;11(12):e1005350. doi: 10.1371/journal.ppat.1005350 (PMC4696832; doi:10.1371/journal.ppat.1005350)

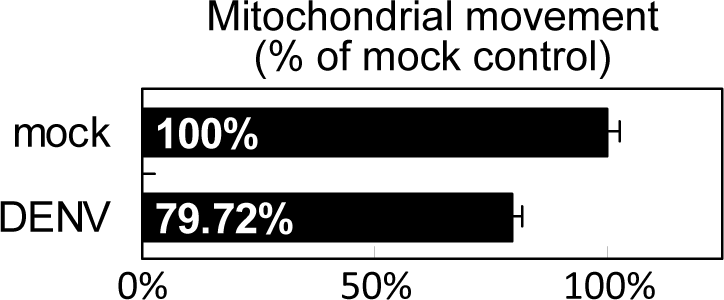

Supplement: S1 Fig — The live confocal microscopy of S1 Movie was composed of photos taken every 0.255 sec. Since the difference between two sequential images within identical field can be subtracted as the movement [61], the sequential mitochondrial movements of DENV-infected cells within 30 sec were analyzed frame-by-frame (117 frames) by Volocity and MetaMorph software and normalized to that of mock. The result is shown as mean ± SD (n = 116, per group) and present in percentage. (TIF) [file ppat.1005350.s003.tif]

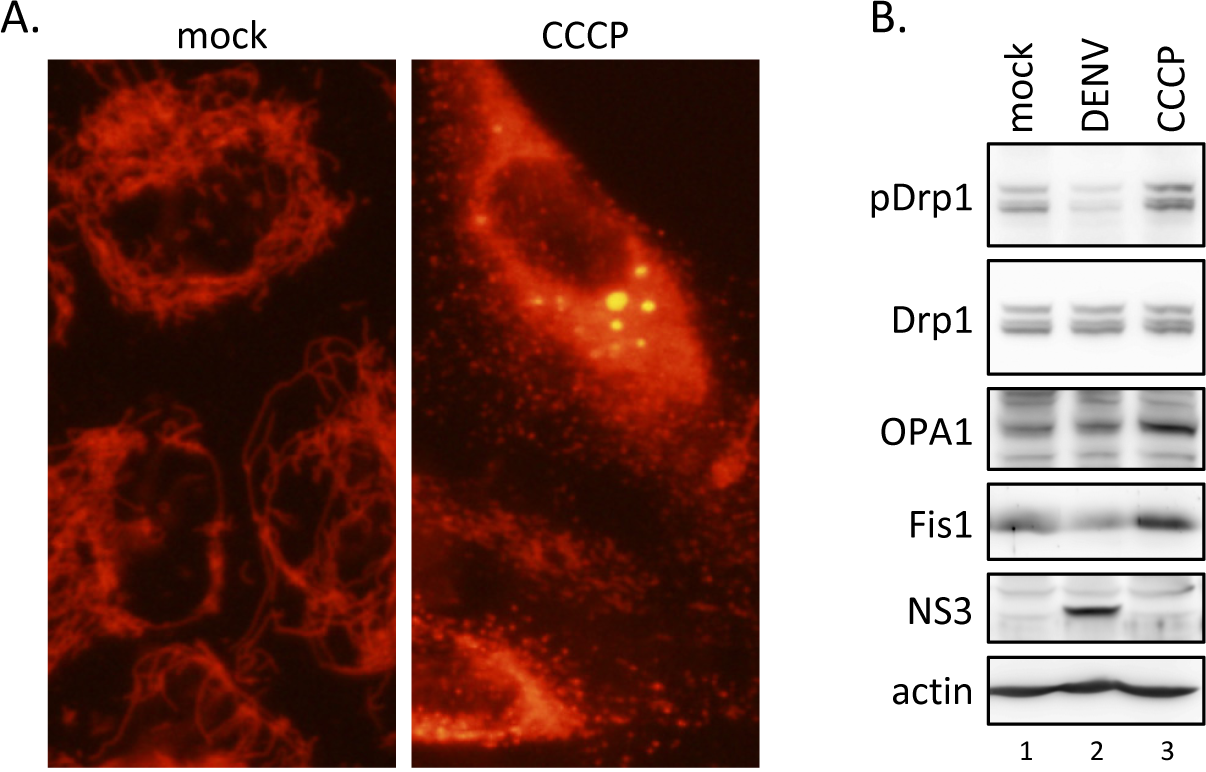

Supplement: S2 Fig — (A and B) A549 cells stably expressing mitoCherry were treated with the ionophore carbonyl cyanide m-chlorophenyl hydrazone (CCCP; 100 μM) or not (mock) for 4 h and analyzed by fluorescent microscopy (A) and by immunoblotting (B). DENV, DENV-infected for 48 h (serotype 2, moi 10). (TIF) [file ppat.1005350.s004.tif]

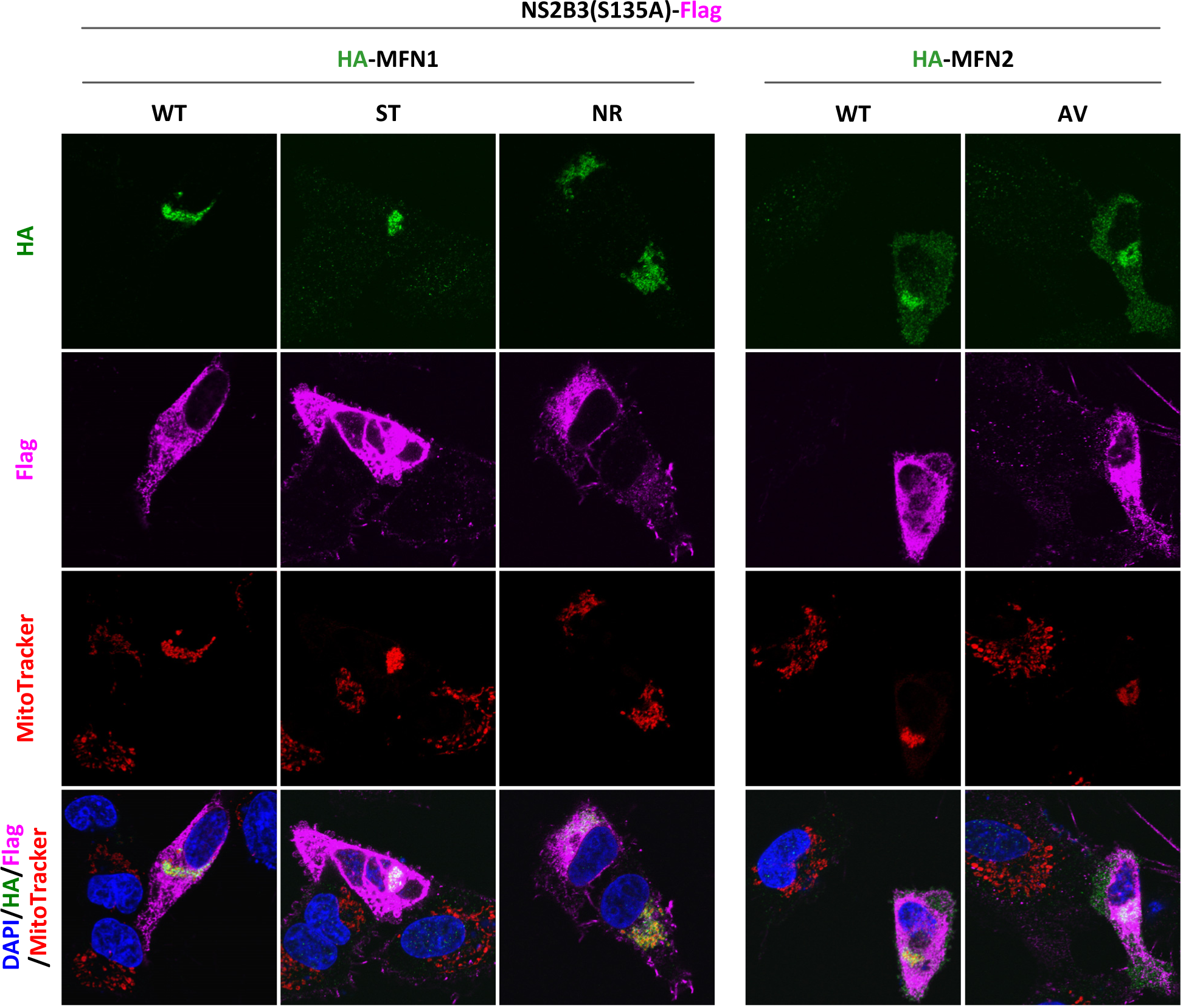

Supplement: S3 Fig — Confocal microscopy of A549 cells cotransfected with Flag-tagged DENV NS2B3(S135A) and the indicated wild-type and mutated MFN constructs for 24 h. Arrows indicate the cells expressing both Flag-tagged DENV protease and HA-tagged MFN. Green: anti-HA; magenta: anti-Flag; red: MitoTracker; blue: DAPI. (TIF) [file ppat.1005350.s005.tif]

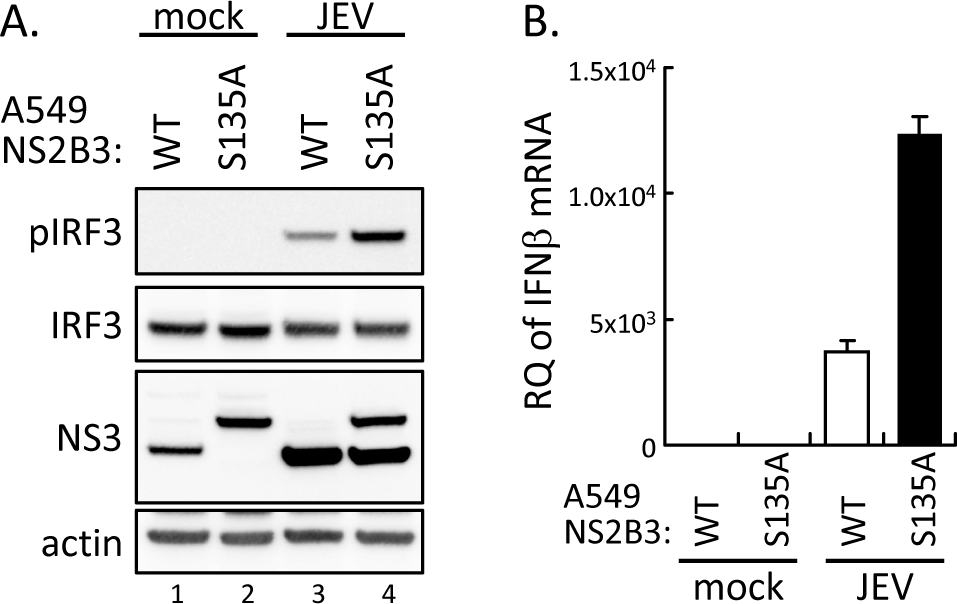

Supplement: S4 Fig — (A and B) A549 cells stably expressing wild-type (WT) or protease-dead (S135A) DENV protease NS2B3 were infected with JEV (moi 10) for 24 h and analyzed by immunoblotting (A) and by RT-qPCR (B). RQ, relative quantification. Note that anti-NS3 antibody recognizes both JEV and DENV NS3. (TIF) [file ppat.1005350.s006.tif]
